# A Tellurium‐Boosted High‐Areal‐Capacity Zinc‐Sulfur Battery

**DOI:** 10.1002/advs.202308580

**Published:** 2024-04-02

**Authors:** Yue Zhang, Amardeep Amardeep, Zhenrui Wu, Li Tao, Jia Xu, Donald J. Freschi, Jian Liu

**Affiliations:** ^1^ School of Engineering, Faculty of Applied Science University of British Columbia Kelowna BC V1V 1V7 Canada; ^2^ Pacific Institute for Climate Solutions and School of Environmental Studies University of British Columbia Kelowna BC V1V 1V7 Canada; ^3^ Fenix Advanced Materials 2950 Highway Drive Trail BC V1R 2T3 Canada

**Keywords:** areal capacity, hybrid electrolyte, hydrogen evolution, redox kinetics, tellurium‐sulfur cathode, zinc‐sulfur battery, Zn dendrites

## Abstract

Aqueous rechargeable zinc‐sulfur (Zn‐S) batteries are a promising, cost‐effective, and high‐capacity energy storage technology. Still, they are challenged by the poor reversibility of S cathodes, sluggish redox kinetics, low S utilization, and unsatisfactory areal capacity. This work develops a facile strategy to achieve an appealing high‐areal‐capacity (above 5 mAh cm^−2^) Zn‐S battery by molecular‐level regulation between S and high‐electrical‐conductivity tellurium (Te). The incorporation of Te as a dopant allows for manipulation of the Zn‐S electrochemistry, resulting in accelerated redox conversion, and enhanced S utilization. Meanwhile, accompanied by the S‐ZnS conversion, Te is converted to zinc telluride during the discharge process, as revealed by ex‐situ characterizations. This additional redox reaction contributes to the S cathode's total excellent discharge capacity. With this unique cathode structure design, the carbon‐confined TeS cathode (denoted as Te_1_S_7_/C) delivers a high reversible capacity of 1335.0 mAh g^−1^ at 0.1 A g^−1^ with a mass loading of 4.22 mg cm^−2^, corresponding to a remarkable areal capacity of 5.64 mAh cm^−2^. Notably, a hybrid electrolyte design uplifts discharge plateau, reduces overpotential, suppresses Zn dendrites growth, and extends the calendar life of Zn‐Te_1_S_7_ batteries. This study provides a rational S cathode structure to realize high‐capacity Zn‐S batteries for practical applications.

## Graphical abstract

This work develops advanced tellurium‐boosted zinc‐sulfur batteries in aqueous and hybrid electrolytes. Due to the remarkable electrical conductivity of tellurium, this battery demonstrates significantly enhanced sulfur utilization, accelerated redox kinetics, and extended life cycles. More importantly, the free‐standing tellurium‐sulfur cathode delivers an excellent areal capacity of up to 5.64 mAh cm^−2^.

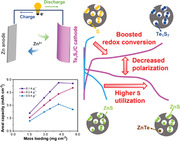

## 1 Introduction

Aqueous zinc‐ion batteries are considered promising energy storage technologies due to their appealing advantages of abundant zinc reserve, environmental friendliness, and good safety.^[^ ^1^ ^]^ To date, a variety of intercalation‐based cathode materials have been attempted in zinc‐ion batteries,^[^ ^2^ ^]^ such as vanadium‐based,^[^ ^3^ ^]^ manganese‐based,^[^ ^4^ ^]^ and Prussian blue analogs.^[^ ^5^ ^]^ However, these cathodes have low specific capacities (<400 mAh g^−1^) that limit the overall energy density of zinc‐ion batteries.^[^ ^6^ ^]^

Since 2020, there has been an increasingly growing research interest in conversion‐based zinc‐sulfur (Zn‐S) chemistry due to its low cost and high theoretical capacity of 1675 mAh g^−1^ (discharge: S + Zn^2+^ + 2e^−^ → ZnS; charge: ZnS − 2e^−^ → S + Zn^2+^).^[^ ^7^ ^]^ Ji et al.^[^ ^8^ ^]^ studied the discharge behavior of Zn‐S primary batteries with different electrolyte salts. They reported the highest capacity of 1668 mAh g^−1^ in the mildly acidic 1 M ZnCl_2_ electrolyte with the highest discharge voltage of ≈0.7 V at 50 mA g^−1^. However, the rechargeability must be solved when aiming at energy storage applications. This work revealed the sluggish reaction kinetics from ZnS to S in the S cathode, resulting in poor cycling stability and fast capacity decline of the Zn‐S batteries.^[^ ^9^ ^]^ Hydrogen evolution reaction (HER) is also a tricky problem for conversion‐type S cathodes because the generated hydrogen from HER side reaction leads to battery volume expansion and even explosion after standing for a long time.^[^ ^10^ ^]^ Inhibition of Zn dendrites is also critical to prolong the battery's lifespan and improve the safety of battery packages.^[^ ^11^ ^]^ Within this context, the most pressing objectives revolve around improving S cathode reversibility, augmenting reaction kinetics, mitigating side reactions, and extending calendar life, all of which are essential for advancing the development of Zn‐S batteries.

In response to the aforementioned challenges encountered in Zn‐S batteries, extensive efforts have been made primarily in the direction of electrolyte optimization to manipulate the cathode‐electrolyte interface,^[^ ^12^ ^]^ including increasing electrolyte concentration^[^ ^13^ ^]^ and adding co‐solvents or additives.^[^ ^10^, ^14^ ^]^ I_2_ additive was used as a Zn‐ion medium to stabilize Zn stripping/plating and reduce the voltage hysteresis of the S cathode in the aqueous Zn‐S battery.^[^ ^15^ ^]^ The as‐constructed Zn‐S battery in the electrolyte of 1 M Zn(CH_3_COO)_2_ and 0.05 wt.% I_2_ delivered a reversible capacity of 1105 mAh g^−1^ with a discharge plateau at 0.5 V. Srinivasan et al. ^[^ ^16^ ^]^ proposed a hybrid aqueous zinc trifluoromethanesulfonate (Zn(CF_3_SO_3_)_2_) electrolyte with ethylene glycol (EG) co‐solvent and zinc iodine (ZnI_2_) additive. EG was found to tune the hydrogen bonding interaction of water molecules through hydroxyl groups and suppress the formation of sulfate and hydrogen sulfide anions. ZnI_2_ was used to enhance the redox conversion kinetics from ZnS to S and alleviate side reactions in the Zn‐S system. Hu et al.^[^ ^9a^ ^]^ employed thiourea (TU) electrolyte additive as a redox mediator to inhibit the generation of SO_4_^2−^ by its strong reactivity with the S atom of ZnS. The introduction of TU also improved the electrochemical dynamics by weakening the Zn‐S bond in the ZnS discharge product. Huang et al.^[^ ^17^ ^]^ developed a deep eutectic solvent (DES) consisting of urea, choline chloride, and acetonitrile to manipulate Zn‐S conversion chemistry. The DES‐based Zn‐S battery possessed better capacity retention (94.48%) in the self‐discharge test compared to Zn‐VO_2_ (47.80%) and Zn‐MnO_2_ (91.57%) batteries.

Rational S cathode structure design is another strategy to achieve high‐energy‐density Zn‐S batteries.^[^ ^18^ ^]^ For example, Lu's research group^[^ ^19^ ^]^ designed atomically Fe sites with Fe‐N_4_ coordination as catalytic sites to promote the redox conversion from ZnS to S during recharging by lowering the ZnS activation energy. Lu et al.^[^ ^20^ ^]^ also disclosed that, instead of Zn depletion on the anode, the aggregation of inactive ZnS was the critical reason for the degradation nature of Zn‐S batteries. They demonstrated a yolk‐shell S@Fe‐PANi cathode to form Zn_x_Fe^II/III^(CN)_6_ redox hotspots, which acted as a cation reservoir to boost the S‐ZnS conversion. Besides, Huang et al.^[^ ^9b^ ^]^ employed ZnS connected by carbon sheath as the cathode, which showed reduced voltage hysteresis and a high specific capacity of 465 mAh g^−1^_ZnS_ (1410 mAh g^−1^_S_) with the help of redox mediator thiourea. Moreover, selenium (Se) or SeS_2_ cathodes were proven to reduce the Zn‐ion diffusion energy barrier and provide efficient Zn‐S or Zn‐Se redox conversions.^[^ ^21^ ^]^ Wang's group^[^ ^22^ ^]^ developed a group of high‐specific‐capacity Se_x_S_y_ cathodes in which the synergistic effect between S and Se facilitated the electrochemical performance of Zn‐S batteries.

Despite these remarkable progresses in the emerging Zn‐S electrochemical system, there are some practical challenges with little discussion.^[^ ^23^ ^]^ First, a majority of reported Zn‐S batteries displayed only specific capacities with low S mass loadings below 3 mg cm^−2^ and areal capacities below 3 mAh cm^−2^ (Table S1, Supporting Information). While these values are crucial for understanding the fundamental performance of the cathodes, they fall short of addressing the critical parameters required for a comprehensive battery assessment, particularly when considering pack‐level applications.^[^ ^6a^ ^]^ Regrettably, previous studies have generally neglected to provide insights into the areal capacities of S cathodes, which are integral for evaluating the practical viability of high‐energy Zn‐S batteries. The S cathode structure design warrants additional attention to propel the continued progress of Zn‐S batteries. Therefore, developing a high‐areal‐capacity S cathode deserves a top priority for high‐energy Zn‐S batteries. Tellurium (Te), with superior electron transport property (2 × 10^2^ S m^−1^) than S (5 × 10^−16^ S m^−1^) and Se (1 × 10^−4^ S m^−1^),^[^ ^24^ ^]^ has proven to accelerate the Li‐S reaction kinetics efficiently with proper addition of Te.^[^ ^25^ ^]^ Te's excellent properties have also inspired growing attention and efforts in the design of Zn‐Te systems.^[^ ^26^ ^]^ Unfortunately, Te‐only cathodes in Zn‐Te batteries usually deliver limited specific capacity below 500 mAh g^−1^ at a low discharge plateau ≈0.6 V.^[^ ^26a–e^ ^]^ Accordingly, integrating both advantages of S and Te is a promising approach to tailor high‐capacity cathodes with fast redox kinetics and S utilization for Zn‐ion batteries. To the best of our knowledge, there has not been a detailed study about the effect of Te in S cathodes for Zn‐S electrochemistry.

**Figure 1:**
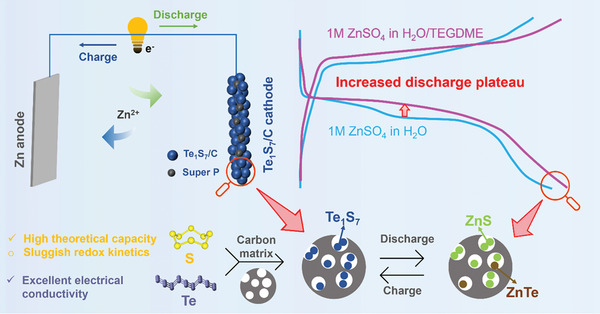
Schematic illustration of the working mechanism in Zn‐Te_1_S_7_ batteries.

This work demonstrated a free‐standing Te‐incorporated S cathode confined into a carbon matrix (Te_1_S_7_/C) for high‐energy Zn‐S batteries. The unique advantages inherent in the Te_1_S_7_ composite structure have been meticulously unveiled through comprehensive characterization and analysis. Te's incorporation was proven to boost the Zn‐S redox conversion, reduce the charge/discharge overpotential, and improve the electrochemical reversibility (**Figure** **1**). Notably, Te within the Te_1_S_7_/C cathodes actively participates in the discharge reaction to form zinc telluride (ZnTe) and therefore contributes substantially to the total discharge capacity. This dual functionality of Te was elucidated through ex‐situ X‐ray diffraction (XRD) and high‐resolution transmission electron microscopy (HRTEM) techniques. The polytetrafluoroethylene (PTFE) based self‐supporting preparation method is an emerging innovative “powder to film” electrode fabrication technology toward the industrialization of next‐generation high‐energy‐density batteries.^[^ ^27^ ^]^ The large‐size free‐standing electrode films can be obtained by the PTFE binder fibrillation,^[^ ^28^ ^]^ which simplifies the manufacturing process, avoids the use of organic NMP solvent, and, most importantly, achieves high mass loading and areal capacity to meet the high‐energy‐density need from battery industry.^[^ ^28^, ^29^ ^]^ Using Zn metal as an anode, the as‐prepared Te_1_S_7_/C cathode exhibits excellent utilization of S and delivers an impressive reversible capacity of 1335.0 mAh g^−1^ at 0.1 A g^−1^ with the cut‐off voltage of 0.1–1.5 V. More importantly, the free‐standing Te_1_S_7_/C cathode exhibited an ultrahigh areal capacity of 5.64 mAh cm^−2^, outperforming most previous S cathodes or intercalation‐type cathodes in zinc batteries. On the other hand, our in‐situ characterizations suggest that a hybrid electrolyte containing tetraethylene glycol dimethyl ether (TEGDME) yields multiple benefits, including mitigating HER, regulating Zn plating/stripping morphology, reducing the polarization of the Zn‐Te_1_S_7_ cell, and ultimately ensuring a long‐cycle‐life Zn‐Te_1_S_7_ battery. These findings are expected to shed light on the development of high‐energy‐density Zn‐S batteries.

The novelty of this work is summarized as follows. First, current Zn‐S or Zn‐Te batteries suffer from limited specific capacity (below 500 mAh g^−1^ for Te‐only electrodes) and unsatisfying areal capacity below 3 mAh cm^−2^. In contrast, our study integrates both advantages of low‐cost S (theoretical capacity: 1675 mAh g^−1^) and high‐electrical‐conductivity Te to fabricate an advanced Te_1_S_7_ cathode with the optimal Te/S atomic ratio of 1:7. This Te‐doped S cathode shows smaller polarization and enhanced reversible discharge capacity than S only and Te only cathode with the same cell configuration (e.g., KB confinement, electrolyte). Second, this work studies the effect of Te content in Te_x_S_y_ cathodes on the electrochemical performance of Zn‐Te_x_S_y_ batteries. 1:7 is the best Te/S atomic ratio among other Te_x_S_y_ cathodes to simultaneously inherit the favorable features of high reversible capacity, improved discharge plateau, and fast redox kinetics. Another highlight is that our free‐standing Te_1_S_7_/C cathodes can achieve adjustable higher mass loadings and areal capacities than the conventional PVDF‐binding Te_1_S_7_/C cathodes. Third, the materials cost of the aqueous Zn‐Te system is significantly reduced by employing substantial low‐cost S. Additionally, despite rapid capacity decay, our electrolyte modification strategy can regulate Zn dendrite growth, eliminate the undesirable HER, as investigated by in‐situ OM and DEMS observations, and therefore extend the lifespan of the Zn‐Te_1_S_7_ battery. Overall, this study about the Zn‐Te_1_S_7_ battery shows advancements in structure design, electrochemical performance, and mechanism analysis.

## 2 Results and Discussions

### 2.1 Cathode Structure Design

**Figure 2:**
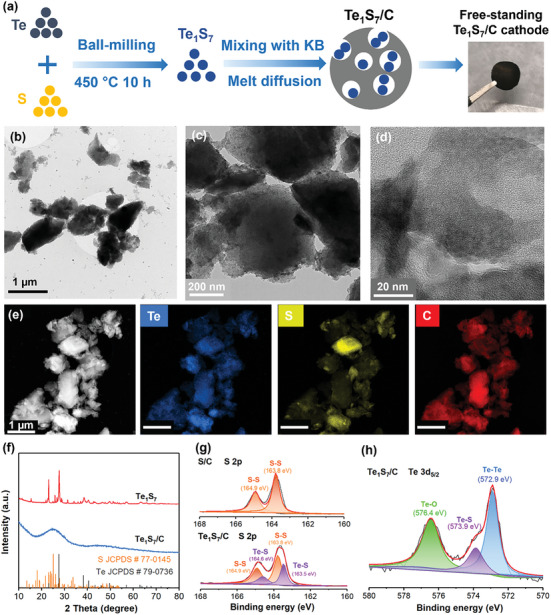
a) Preparation scheme of Te_1_S_7_/C cathodes, b) TEM, c,d) HRTEM, e) HAADF images and elemental mapping of Te_1_S_7_/C composites, f) XRD patterns of Te_1_S_7_ and Te_1_S_7_/C, g) XPS S 2p spectra of S/C and Te_1_S_7_/C cathodes, and h) Te 3d_5/2_ XPS spectra of the Te_1_S_7_/C cathode.

In this work, Te and S powders were mixed with a Te/S molar ratio of 1:7 by high‐energy ball‐milling and subsequent heating treatment to form the Te_1_S_7_ compound, as illustrated in **Figure** **2**a and Figure S1a (Supporting Information). Then Te_1_S_7_ was loaded and infused into the micro/mesopores of KB carbon through a melt‐diffusion treatment (480 °C for 10 h) (Figure S1b, Supporting Information). KB possessed a large pore volume (2.6 cm^3^ g^−1^) and surface area (1382.42 m^2^ g^−1^) with numerous mesopores centered at 4 nm (Figure S2, Supporting Information), offering sufficient loading sites for confining Te_1_S_7_ active materials. Te_1_S_7_/C, S/C, and Te/C materials were fabricated using the same melt diffusion method, where the active materials (Te_1_S_7_, S, Te) were melted at high temperatures above their individual melting points, and then were infused into porous carbon by capillary forces.^[^ ^30^ ^]^ As the temperature gradually cooled, the active materials solidified within the host carbon structure. Specifically, the heating temperature used for Te_1_S_7_/C, S/C, and Te/C materials in this study were 160, 480, and 480 °C, respectively, based on the melting points of S (115.21 °C) and Te (449.51 °C), as well as previous relevant studies.^[^ ^30^, ^31^ ^]^ Te_1_S_7_ content in Te_1_S_7_/C and S content in S/C are 47 wt.% and 54 wt.%, respectively, as confirmed by thermogravimetric analysis (TGA) (Figure S3, Supporting Information). PTFE, copolymers of tetrafluoroethylene and other monomers (such as ethylene), is a fibrillation‐type binder that is different from conventional PVDF binder. A key characteristic of PTFE is the loosely arranged crystal structure, attributed to the relatively low van der Waals force between the PTFE molecular chains.^[^ ^29a^ ^]^ When the temperature is higher than 19 °C, the crystal structure of PTFE changes from triclinic to hexagonal, resulting in softened molecular chains (called fibrils) that can be pulled out by a small shearing force.^[^ ^27a^ ^]^ Consequently, the fibrillation of PTFE forms a matrix to support electrode particles. In our study, a dough‐like Te_1_S_7_/C electrode was obtained after manually grinding the mixture of Te_1_S_7_/C, super P, and PTFE binder for 20 min. This dough was subsequently rolled into a piece of free‐standing electrode (Figures S4 and S5, Supporting Information) benefiting from the PTFE binder fibrillation.

Figure 2b,c show transmission electron microscopy (TEM) images of the Te_1_S_7_/C composite at the nanometer scale, and these Te_1_S_7_/C particles had irregular shapes with an average length below 1 µm. Notably, no apparent lattice fringe was observed from the HRTEM image (Figure 2d), implying an amorphous structure of Te_1_S_7_ embedded in the carbon matrix. Moreover, Te and S elements were uniformly distributed in the KB carbon matrix, as seen in Figure 2e.

X‐ray diffraction (XRD) tests of Te_1_S_7_ and Te_1_S_7_/C samples were performed to analyze their crystal structure. As shown in Figure 2f, the TeS compound displayed groups of characteristic peaks of S and Te, suggesting the successful incorporation of Te and S after ball‐milling and heat treatment. However, the Te_1_S_7_/C only had a weak broad peak at 25° after loading TeS materials into carbon at 480 °C. The disappearance of S or Te peaks indicated an amorphous Te_1_S_7_ structure,^[^ ^31^, ^32^ ^]^ which agreed well with the HRTEM finding (Figure 2d). X‐ray photoelectron spectroscopy (XPS) analysis of the Te_1_S_7_/C composite was carried out to validate the possible Te‐S compound. As seen in Figure 2g, a pair of peaks assigned to the S─S bond was found from both S/C and TeS/C cathodes at 164.9 and 163.8 eV, respectively. Another pair of peaks at 164.6 and 163.5 eV were deconvoluted, corresponding to the Te─S bond in the TeS/C composite.^[^ ^25c,d^ ^]^ XPS Te 3d_5/2_ spectra (Figure 2h) demonstrated three significant peaks at 576.4, 573.9, and 572.9 eV, which can be ascribed to the Te─O, Te─S, and Te─Te bonds, respectively. The existence of the Te─S bond revealed that the TeS compound was not a simple physical mixture of Te and S elements. The interaction between Te and S atoms was formed after the heat treatment, and it was expected to boost the Zn‐S electrochemical conversion with Te as the redox mediator. We infer that Te and S are chemically bonded instead of physically mixed after high‐energy ball‐milling and subsequent high‐temperature treatment (450 °C for 10 h). It was reported that Te─S covalent bonds in the molten Te_x_S_y_ mixtures could be formed at high temperatures.^[^ ^33^ ^]^ In addition to XPS, another supporting information about Te─S bonds is the slight shift of characteristic Te peak from 122 cm^−1^ for pure Te to 126 cm^−1^ for Te_1_S_7_ in Raman spectra based on our recent study.^[^ ^31b^ ^]^ The ball‐milled Te&S mixture without heat treatment retains all characteristic peaks of Te and S without apparent peak shift. Besides, the existence of Te‐S bonds in Te_x_S_y_ compounds can help uniform Te distribution and accelerate the Li‐S electrochemical activity.^[^ ^25b,c^ ^]^

### 2.2 Electrochemical Performance

**Figure 3:**
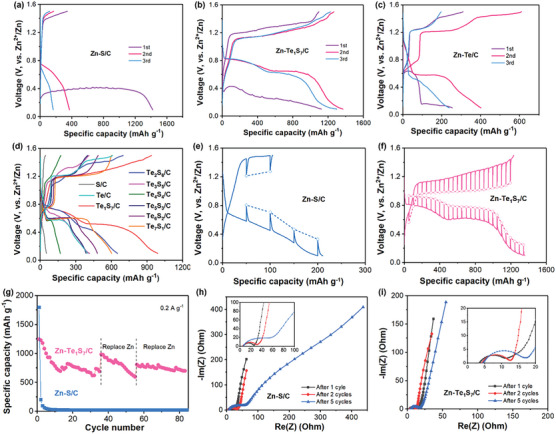
Galvanostatic charge–discharge profiles of a) Zn‐S/C, b) Zn‐Te_1_S_7_/C, c) Zn‐Te/C at 0.1 A g^−1^, and d) Zn‐Te_x_S_y_/C batteries at 0.2 A g^−1^, GITT plots of e) Zn‐S/C and f) Zn‐Te_1_S_7_/C batteries in the 2nd cycle, g) cycling performance of Zn‐S/C and Zn‐Te_1_S_7_/C batteries at 0.2 A g^−1^ with a cut‐off voltage of 0.1–1.5 V, Nyquist plots of h) Zn‐S/C and i) Zn‐Te_1_S_7_/C batteries after 1, 2, and 5 cycles.

The as‐prepared free‐standing Te_1_S_7_/C cathode was assembled in a coin cell with Zn foil as the anode to test its electrochemical properties. S/C cathodes were also prepared and tested for comparison. **Figures** **3**a,b and Figure S6 (Supporting Information) show CV plots and galvanostatic charge/discharge profiles of Zn‐S/C and Zn‐Te_1_S_7_/C batteries in the initial three scans. As seen in Figure S6a (Supporting Information), the Zn‐S cell had a strong reduction peak corresponding to the S‐ZnS conversion,^[^ ^20^ ^]^ but the oxidation peak was weak, implying sluggish kinetics when ZnS was converted to S. Moreover, the peak current was drastically lower after each scan, suggesting the irreversibility feature of Zn‐S electrochemistry. In contrast, the Zn‐Te_1_S_7_/C battery demonstrated highly overlapped CV profiles (Figure S6b, Supporting Information), indicating excellent electrochemical reversibility of Zn‐S conversion with the assistance of Te. Moreover, the Zn‐S/C battery delivered a low charge capacity and Coulombic efficiency (CE) of 24.1% despite a high discharge capacity of 1420.2 mAh g^−1^ in the initial cycle at the current density of 0.1 A g^−1^ (Figure 3a). Afterward, the discharge capacity was reduced to 370.6 and 164.5 mAh g^−1^ in the 2nd and 3rd cycles, respectively. In comparison, the Zn‐Te_1_S_7_/C demonstrated an ultrahigh initial CE of 97.5% and stable discharge capacities of 1122.4, 1291.5, and 1224.4 mAh g^−1^ in the first three cycles (Figure 3b). As for the Te/C cathode, it exhibits specific capacities of 253.7, 400.3, and 237.0 mAh g^−1^ in the initial 3 cycles, respectively (Figure 3c). The suddenly shortened discharge plateau at 0.6 V and reduced capacity in the 3^rd^ cycle suggest poor reversibility of Te only cathode. Besides, the 2^nd^‐cycle charge/discharge profiles of other Te_x_S_y_/C cathodes are shown in Figure 3d. It is observed that the discharge plateaus at ≈0.7/0.5 V are becoming shortened, and less specific capacities (989.1, 650.1, 415.4, and 168.3 mAh g^−1^) are delivered with the increase of Te content (from Te_1_S_7_ to Te_4_S_4_). As Te content in Te_x_S_y_ goes up further, the flat plateau at 0.6 V extends and contributes to higher capacities (385.5, 481.2, and 600.8 mAh g^−1^). The superior discharge capacity of the Te_1_S_7_/C cathode than that of S/C, Te/C, and other Te_x_S_y_/C cathode reveals the better utilization of active materials, faster ions/electrons transport, and more reversible ZnS‐S conversion with the optimal Te/S ratio of 1:7.

Additional two porous carbons, mesoporous BP2000 and micropores‐dominated activated carbon ASAC25, were also employed as host materials for Te_1_S_7_. Their electrochemical performance was tested and evaluated for comparison. As seen in Figure S7 (Supporting Information), the ASAC25 carbon shows the lowest specific capacities over 35 cycles, contributing to only ≈200 mAh g^−1^ capacity at each discharge plateau (0.7/0.5 V) in the 2nd voltage‐capacity profile. The BP2000 carbon initially displays the highest initial capacity of 1298.3 mAh g^−1^ but experiences a gradual capacity decrease, remaining only 432.4 mAh g^−1^ after 35 cycles. In comparison, KB demonstrates a superior capacity retention (691.9 mAh g^−1^ in the 35th cycle) than ASAC25 and BP2000, despite a fast capacity decay in the first 10 cycles.

Regarding the selection of electrolyte salt, this work also employed aqueous 1 M Zn(OTf)_2_ electrolyte to study the effect of zinc salt on the electrochemical performance of Zn‐Te_1_S_7_/C batteries. As seen in Figure S8 (Supporting Information), the discharge plateau at 0.6 V becomes shorter and contributes to a relatively low specific capacity of 864.8 mAh g^−1^ in the 5th cycle, compared to the discharge capacity of 1002.7 mAh g^−1^ delivered by the Zn‐Te_1_S_7_/C cell with ZnSO_4_ salt at the same voltage range of 0.1–1.5 V (Figure 3b).

For comparison, the Te_1_S_7_/C materials are also mixed with Super P and PVDF to form a slurry, which is cast onto Ti foil and dried overnight. The as‐obtained Te_1_S_7_/C cathode exhibits fluctuated voltage‐capacity profiles (Figure S9, Supporting Information) even with a low mass loading of 0.6 mg cm^−2^ at the current density of 0.1 A g^−1^, indicating poor interfacial contact between cathode particles. The voltage‐capacity profiles of batteries can indicate various factors such as uneven discharge, temperature variation, electrode degradation, and internal resistance variation.^[^ ^34^ ^]^ The effect of temperature could be minimized as all battery tests were conducted under consistent temperature conditions. The fluctuating voltage profiles in the initial cycles in Figure S7 (Supporting Information) imply non‐uniform movement of Zn‐ions in the Te_1_S_7_/C cathode (prepared by PVDF binder, cast onto Ti foil), potentially due to irregular internal resistance during charge/discharge process. Similar voltage fluctuations are also noticeable in the first cycle of the free‐standing S/C cathode (refer to Figure 3a), which might be a result of the uneven discharge status among S/C particles. These slight voltage fluctuations may also indicate the electrode's structural reconstruction during the electrochemical activation.^[^ ^35^ ^]^ The self‐adaptability behavior of electrode materials under thermodynamic force, due to the high charge density of Zn^2+^, is observed in a few Zn‐ion batteries, which is affected by several factors, such as particle size, electrode structure, and electrolyte component.^[^ ^35^ ^]^

**Figure 4:**
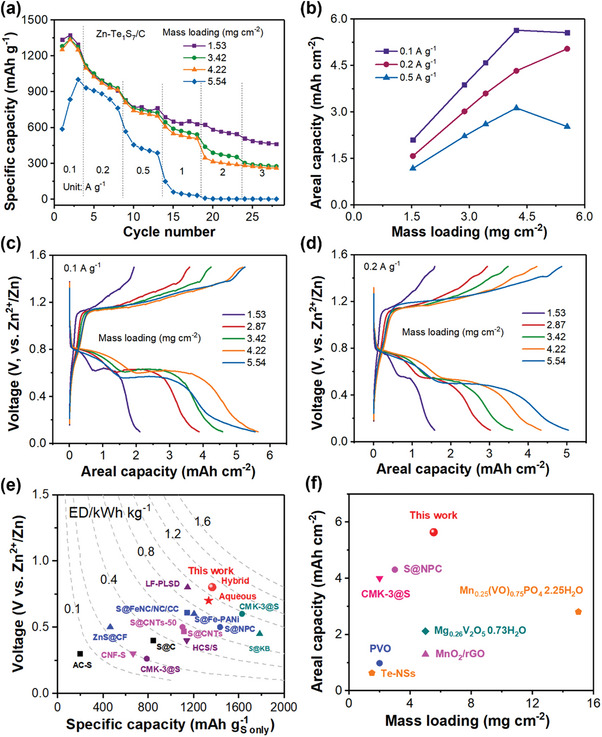
a) Rate performance of Te_1_S_7_/C cathodes with various mass loadings, b) areal capacities versus mass loadings at different current densities for Te_1_S_7_/C cathodes, charge/discharge profiles of Te_1_S_7_/C cathodes at c) 0.1 A g^−1^ and d) 0.2 A g^−1^, e) comparison of specific capacity, voltage and energy density between previous S cathodes and this work in aqueous Zn‐S batteries, and f) areal capacity and mass loading comparison with other cathodes in Zn‐ion batteries.

In comparison, our PTFE‐based free‐standing Te_1_S_7_/C cathode can deliver more stable specific capacities and areal capacities at the mass loading range of 1.53–4.22 mg cm^−2^ (**Figure** **4**a,b). In addition, increasing the working temperature to 40 °C is an effective way of uplifting the discharge plateau and reducing the battery polarization for the Zn‐Te_1_S_7_/C battery (Figure S10, Supporting Information).

The redox kinetics of Zn‐S/C and Zn‐Te_1_S_7_/C batteries were tested and evaluated by the galvanostatic intermittent titration technique (GITT) and electrochemical impedance spectroscopy (EIS) methods. Figure 3e,f illustrate the different electrochemical behavior of both cells from GITT measurement. The Zn‐S/C battery showed short discharge/charge slopes with large polarization both in dynamic (solid lines) and transient curves (circle points) (Figure 3e). The Zn‐Te_1_S_7_/C battery exhibited flat discharge plateaus at ≈0.8 and 0.6 V (Figure 3f), corresponding to the formation of intermediates (polysulfotellurides) and final discharge products (ZnS/ZnTe).^[^ ^22^, ^25^ ^]^ Moreover, the Te‐enhanced Zn‐S chemistry possessed smaller polarization, implying a lower energy barrier for Zn‐ion migration in the S cathode. The reaction resistance during the discharge/charge process was also calculated based on GITT profiles.^[^ ^31b^ ^]^ As seen in Figure S11 (Supporting Information), the dynamic reduction resistance of the Zn‐Te_1_S_7_/C battery was only one‐fifth of that of the Zn‐S/C battery, with the discharge depth increasing. The two slight growths in the Zn‐Te_1_S_7_/C battery corresponded to the completion of the discharge plateaus and might be attributed to the volume change of active materials. Notably, the oxidation resistance during the charging process kept growing with the charge depth, suggesting slower charge kinetics than discharge kinetics when ZnS/ZnTe was converted back to Te_1_S_7_ materials. The superior redox kinetics in the Zn‐Te_1_S_7_/C battery benefited from the effective molecular regulation by the high‐electrical‐conductivity Te atoms.

The cycling stability of the Zn‐S/C and Zn‐Te_1_S_7_/C batteries were tested at 0.2 A g^−1^ and plotted in Figure 3g. The Zn‐S/C battery delivered an ultralow capacity of 203.5 mAh g^−1^ in the 2nd cycle and then declined rapidly to 31.5 mAh g^−1^ in the 10th cycle. As for the Zn‐Te_1_S_7_/C battery, the initial capacity was as high as 1185.9 mAh g^−1^ and then the discharge capacity dropped to 785 mAh g^−1^ in the 10th cycle. Afterward, the remaining reversible specific capacity was stabilized at ≈700 mAh g^−1^ until a sudden short circuit after the 35th cycle. However, the battery kept running after replacing a fresh Zn anode and terminated again after another 20 cycles. The rapid capacity fading of the aqueous Zn‐Te_1_S_7_/C battery could possibly originate from the shuttle effect caused by the dissolution of polysulfides or polytellurides intermediates into the aqueous solution (possible redox reaction: S_8_ → S_x_^2−^ → S^2−^ or Te → Te_x_^2−^ → Te^2−^).^[^ ^16^, ^26^ ^]^ It should be noted that Zn striping/plating continuously occurred on the anode side during the long reduction/oxidation cycles. The severe Zn dendrites and undesired HER side reaction on the anode side mainly cause the short life cycle. Therefore, both Zn anode and TeS cathode require suitable design to enable a long‐calendar‐life and high‐capacity Zn‐Te_1_S_7_/C battery, which will be studied and discussed in the later Section 2.4.

The elevated discharge plateau and reduced polarization by Te doping indicate faster redox kinetics in Zn‐S batteries. A deeper understanding at the atomic level sheds light on this phenomenon. Encouragingly, our recent exploration in Li‐Te_x_S_y_ battery has revealed that Te incorporation in the S_8_ structure reshapes the Li‐S electrochemistry, resulting in the more delocalized charge‐density differences around the Te atoms in Li_16_Te_1_S_7_ compared to site‐equivalent S atoms in Li_2_S.^[^ ^31b^ ^]^ Te doping also pushes the S 2p states closer to the Fermi level and improves the electrical conductivity in Li_16_Te_1_S_7_. Consequently, the energy barrier for Li‐ion migration is lowered, leading to accelerated lithiation/delithiation kinetics. Specifically, Te_1_S_7_ possesses the lowest energy profile of Li‐ion migration pathways among other Te_x_S_y_ composites. Drawing inspiration from this Li‐Te_1_S_7_ battery scenario, we speculate a similar mechanism for Zn‐Te_1_S_7_ electrochemistry. Te incorporation improves electrical conductivity and decreases the energy barrier for Zn‐ion migration, thus facilitating the reaction kinetics and elevating the discharge plateau. Nevertheless, further study of the mechanism is still required to prove this working mechanism.

In terms of the noticeable fast capacity fading of the S/C cathode in the initial 5 cycles in Figure 3g, we have collected Nyquist plots of both Zn‐S/C and Zn‐Te_1_S_7_/C batteries after 1, 2, and 5 cycles at 0.2 A g^−1^ to track the resistance change of both samples. As seen in Figure 3h,i, Figures S12 and S13, and Table S2 (Supporting Information), the Zn‐S/C battery has significantly increased charge transfer resistance R_ct_ (from 20.33 Ω after the 1st cycle to 51.3 Ω after the 5th cycle) and interfacial resistance (or solid electrolyte interphase resistance R_SEI_) (from 9.98 Ω after the 1st cycle to 404 Ω after the 5th cycle). In contrast, the Zn‐Te_1_S_7_/C battery keeps relatively stable R_ct_ (11.43 Ω) and R_SEI_ (10.47 Ω) after the initial 5 cycles, implying the superior structural or interfacial stability of Te_1_S_7_/C in the first 5 cycles than the S/C cathode.

The rate capability test of the free‐standing Te_1_S_7_/C cathodes was carried out at various current densities of 0.1–3 A g^−1^. As shown in Figure 4a, the Te_1_S_7_/C cathodes with mass loadings of 1.53, 3.42, and 4.22 mg cm^−2^ delivered close specific capacities of 1373.4/1339.9/1335.0, 1028.7/1051.1/1024.0, and 769.4/762.7/741.9 mAh g^−1^ at the current densities of 0.1, 0.2, and 0.5 A g^−1^. With the further current increase, the cathode with 1.53 mg cm^−2^ demonstrated the highest capacities of 650.7, 582.5, and 487.3 mAh g^−1^ at 1, 2, and 3 A g^−1^, respectively. The Te_1_S_7_/C cathode with higher active materials mass loadings (3.42 and 4.22 mg cm^−2^) had lower specific capacities of 590.4/550.1, 389.4/315.9, and 290.9/275.4 mAh g^−1^ under high current densities of 1, 2, and 3 A g^−1^, respectively. Specifically, the increased mass loading of 5.54 mg cm^−2^ leads to the drastically lowest specific capacities compared to other cathodes with lower mass loadings at the same testing range of 0.1–3 A g^−1^.

Despite similar specific capacities at 0.1–0.5 A g^−1^, these Zn‐Te_1_S_7_ batteries exhibited excellent areal capacities (Figure 4b), which were proportion to the mass loadings (below 4.22 mg cm^−2^). As the mass loading of the Te_1_S_7_/C cathode went up from 1.53 to 4.22 mg cm^−2^, the corresponding areal capacity increased significantly from 2.1 to 5.64 mAh cm^−2^ at 0.1 A g^−1^, which outperformed other cathodes in aqueous rechargeable Zn batteries (Table S1, Supporting Information). However, higher mass loading (5.54 mg cm^−2^) caused a slight decrease in areal capacity (5.56 mAh cm^−2^) and a sharp reduction in specific capacity (835.9 mAh g^−1^), which might be due to the large charge transfer resistance.^[^ ^21c^ ^]^ Figure 4c,d, and Figure S14 (Supporting Information) illustrate the charge/discharge profiles of the Zn‐Te_1_S_7_/C batteries with varying Te_1_S_7_ mass loading. It is observed that, with mass loading growing (below 4.22 mg cm^−2^), the voltage‐capacity profile possessed a higher discharge plateau and lower charge plateau, suggesting the reduced polarization for the reversible S‐ZnS conversion. The extended discharge plateaus at 0.8/0.6 V versus Te_1_S_7_ content implied improved materials utilization. Specifically, the Te_1_S_7_/C cathode with a high loading of 5.54 mg cm^−2^ had the largest charge‐discharge voltage difference, indicating the slower reaction kinetics in high‐loading Te_1_S_7_/C cathodes when Zn‐ions were inserted into or exacted from Te_1_S_7_/C materials.

In summary, Figure 4a,b depict the relationship between the electrochemical performance of the Zn‐Te_1_S_7_/C battery and the mass loading of Te_1_S_7_ active materials. The Te_1_S_7_/C cathodes with varied mass loadings (1.53, 3.42, and 4.22 mg cm^−2^) deliver close specific capabilities at small current densities of 0.1, 0.2, and 0.5 A g^−1^, indicating the remarkable utilization of Te_1_S_7_ active materials under low current densities. Therefore, the corresponding areal capacities also increase versus the mass loadings under those current conditions. However, with further increase of current density, redox kinetics are the primary determining factor of the S‐ZnS conversion, thus leading to decreased S utilization. In that case, higher mass loadings limit the complete transformation between Te_1_S_7_ and ZnS/ZnTe and result in the dropped specific capacities with varying loadings. It is also found that excess mass loading (5.54 mg cm^−2^) cannot achieve the maximum utilization of Te_1_S_7_ and causes the lowest specific capacity even at 0.1 A g^−1^. Accordingly, it is concluded that the Zn‐Te_1_S_7_/C battery possessed high specific capacity (1335.0 mAh g^−1^) and remarkable areal capacity (5.64 mAh cm^−2^) when the mass loading is as high as 4.22 mg cm^−2^, while excess active materials content slowed down the ion/electron transport, thus causing decreased capacity and inferior materials utilization. More importantly, this Zn‐Te_1_S_7_/C battery showed a competitive energy density of 934.6 Wh kg^−1^ based on the TeS materials among the reported Zn‐S batteries (Figure 4e) ^[^ ^9^, ^10^, ^15^, ^16^, ^17^, ^19^, ^20^ ^]^ and exceptional areal capacity of 5.64 mAh cm^−2^ compared to the intercalation‐type cathodes in Zn‐ion batteries (Figure 4f),^[^ ^36^ ^]^ as summarized in Table S1 (Supporting Information).

### 2.3 Reaction Mechanism

**Figure 5:**
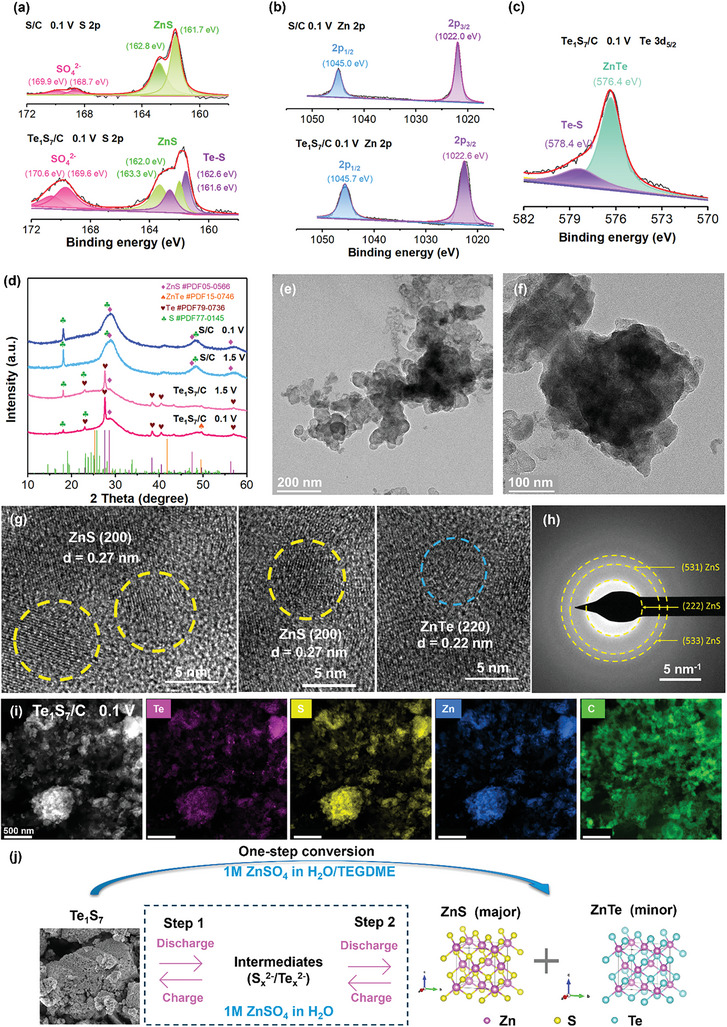
a) XPS S 2p, b) Zn 2p, and c) Te 3d_5/2_ spectra of S/C and Te_1_S_7_/C cathodes upon discharged to 0.1 V, d) XRD patterns of S/C and Te_1_S_7_/C cathodes at fully discharged (0.1 V) and charged (1.5 V) states, e,f) TEM, g) HRTEM, h) SAED, i) HADDF and elemental mapping of the fully discharged Te_1_S_7_/C cathode, j) proposed reaction pathways of Zn‐Te_1_S_7_ batteries in aqueous and hybrid electrolytes.

To determine the possible redox conversion in the Zn‐S system, the fully charged/discharged Zn‐S and Zn‐Te_1_S_7_ cells were disassembled for further investigation. As shown in **Figure** **5**a, the S/C cathode at 0.1 V had a pair of peaks located at 162.8 and 161.7 eV corresponding to the formation of ZnS, and another pair of peaks at 169.9 and 168.7 eV that can be assigned to SO_4_^2−^ from the electrolyte.^[^ ^21b^ ^]^ As for the discharged Te_1_S_7_/C cathode, the pair of peaks located at 163.3 and 162.0 eV can be assigned to ZnS, while the other pair of peaks at 162.6 and 161.6 eV can be attributed to Te‐S bonds in Te_1_S_7_. The Te_1_S_7_/C cathode at 0.1 V also possessed higher binding energies for Zn 2p spectra (1045.7 and 1022.6 eV for Zn 2p_1/2_ and Zn 2p_3/2_, respectively) than the S/C cathode (1045.0 and 1022.0 eV for Zn 2p_1/2_ and Zn 2p_3/2_, respectively), as illustrated in Figure 5b. Additionally, the discharged Te_1_S_7_/C cathode was found to have a prominent peak that corresponds to the formation of ZnTe upon the completion of the discharge process ^[^ ^26a^ ^]^ in XPS Te 3d_5/2_ spectra (Figure 5c).

As seen in Figure 5d, both charged S/C and Te_1_S_7_/C samples demonstrate one characteristic peak of S at 18.1°, corresponding to the (1 2 0) planes of S according to PDF #77‐0145. Besides, the S/C sample displayed one broad peak at 28.1° and one small peak at 48.4°, which can be attributed to the (1 1 3) and (3 5 0) planes of S, respectively. A prominent peak at 27.6° and a couple of minor peaks at 38.3°, 40.5°, and 56.9° suggest the conversion from ZnTe to Te. As for the discharge process, the noticeable peak at 28.6° can be indexed to the (1 1 1) plane of ZnS (PDF# 05–0566), indicating the transformation from S to ZnS. Meanwhile, a weak small peak at 49.5° appears, which might be ascribed to the (3 1 1) plane of ZnTe (PDF#15‐0746). It should be noted that these two cathode samples involve substantial amorphous carbon (KB or Super P), which could be one possible reason for the weak XRD signals of active materials. The noticeable peaks originating from S and Te in the discharged Te_1_S_7_/C sample indicate that a small amount of Te_1_S_7_ materials exist in the discharged cathode. Based on these findings, ZnS was the primary discharge product of Te_1_S_7_/C and S/C cathodes. Meanwhile, the small amount of Te in Te_1_S_7_/C was reduced to ZnTe as the co‐discharge product.

Moreover, SEM, HRTEM, and SAED techniques were employed to investigate the morphology and structure change of the discharged S/C and Te_1_S_7_/C cathodes. The discharged Te_1_S_7_/C cathodes seemed to have more compact and regulated surface morphology, while the charging effect was significant in the S/C cathode (Figure S15, Supporting Information). As shown in Figure S16 (Supporting Information), the discharged S/C cathode had a small crystalline area and a majority of disordered structure that can be ascribed to amorphous carbon (KB host or conductive carbon Super P). The lattice fringes, as highlighted, corresponded to the interplanar distance of 0.31 nm, originating from the (111) plane of ZnS. The Te_1_S_7_/C cathode displayed a larger crystalline area with lattice fringes in Figure 5e–g, implying that more crystalline ZnS materials were generated from the Te_1_S_7_/C structure through the Zn‐S redox conversion. It is noteworthy that the lattice fringes with interplanar distances of 0.22 and 0.27 nm were identified, which were correlated to the (2 2 0) plane from ZnTe and the (2 0 0) plane from ZnS. The three significant diffraction rings in Figure 5h and Figure S16c (Supporting Information) were assigned to the (533), (531), and (222) planes of ZnS, which were both detected in the discharged S/C and Te_1_S_7_/C cathodes. The uniform elemental distribution was observed in both Te_1_S_7_/C and S/C cathodes (Figure 5i; Figure S17, Supporting Information).

**Figure 6:**
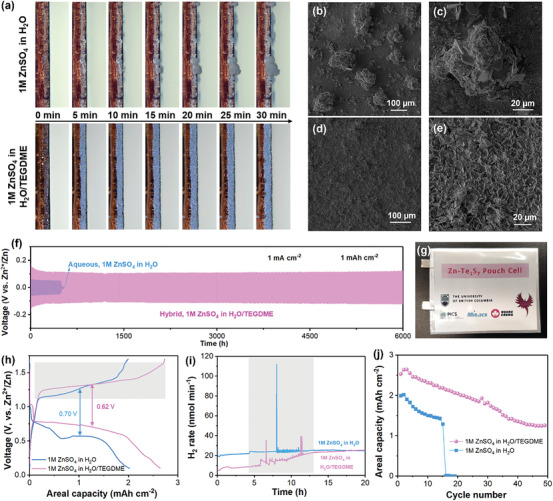
a) Real‐time captured images of Zn^2+^ plating in aqueous (1 M ZnSO_4_ in H_2_O) and hybrid (1 M ZnSO_4_ in H_2_O/TEGDME) electrolytes, SEM images of Zn surface after 5 cycles of Zn plating/stripping in b,c) aqueous and d,e) hybrid electrolytes, f) cycling performance of Zn symmetrical cells in both aqueous and hybrid electrolytes, g) digital photo of a Zn‐Te_1_S_7_ pouch cell (electrode area 3 cm × 3 cm) with the hybrid electrolyte, h) charge–discharge curves of Zn‐Te_1_S_7_/C batteries with aqueous and hybrid electrolytes, and i) the corresponding in situ monitored H_2_ evolution curve, and j) areal capacities over cycles of the Zn‐Te_1_S_7_/C cells in both electrolytes with a cut‐off voltage of 0.1–1.7 V.

The energy storage mechanism proposed in this work (Figure 5j) is built upon insights gathered from various characterization techniques working together. Collectively, the consistent findings from ex‐situ XRD, TEM, and XPS reveal that the Te_1_S_7_ active materials were ultimately converted to ZnS (primary discharge product) and ZnTe when the Zn‐Te_1_S_7_/C cell was discharged to 0.1 V. In particular, the stepwise discharge profile in Figure 3 implies the generation of intermediate products (S_x_^2−^/Te_x_^2−^) in the aqueous electrolyte of 1 M ZnSO_4_ in H_2_O, whereas the single plateau in 1 M ZnSO_4_ in H_2_O/TEGDME (**Figure** **6**) indicates one‐step redox conversion (Te_1_S_7_ + 8Zn^2+^ + 16e^−^ ↔ 7ZnS + ZnTe). Overall, the incorporation of Te plays a bifunctional role in the Zn‐S electrochemistry manipulation. The electrochemically active Te can boost Zn‐S chemistry, facilitate the Zn‐ion conduction and electron transport, and enhance the utilization of S‐active materials. Meanwhile, the unique Zn‐Te redox conversion can generate the ZnTe discharge product via a two‐electron reaction and contribute to the total capacity. This detailed analysis aims to deepen the fundamental understanding of Te‐enhanced Zn‐S batteries. Our findings offer a facile strategy to boost Zn‐S electrochemistry through molecular regulation and enable high‐areal‐capacity electrodes toward practical applications.

### 2.4 Zn Anode Modification

Although the Te_1_S_7_/C cathode demonstrated significantly improved electrochemical performance (capacity, polarization, kinetics) than the S/C cathode, some issues still have not been solved. As seen in Figure 3h, the Zn‐Te_1_S_7_/C cell showed a fast capacity decline to 700 mAh g^−1^ and suddenly stopped working after 35 cycles (500 h). Replacing fresh Zn anode was one solution to extend the battery's life cycle, but it is not a facile approach in practical applications. In order to sort out the failure mechanism of Zn‐Te_1_S_7_/C batteries in aqueous electrolytes, the in‐situ optical microscopy (OM) technique was applied to observe the zinc dendrites growth on the Zn surface under a current density of 2 mA cm^−2^. As illustrated in Figure 6a, the cross‐section of Zn foil was becoming rough, with more and more large Zn dendrites irregularly growing during Zn plating. For further observation by OM, numerous Zn dendrites shown in black color above 50 µm diameter were formed non‐uniformly on the Zn surface (Figure 6b,c; Figure S18a,b, Supporting Information). These continuously generated Zn dendrites were prone to piercing the glass fiber separator and caused an abrupt internal short circuit, thus shortening the battery lifetime. Herein, a hybrid electrolyte with organic TEGDME as a co‐solvent is used to manipulate the Zn deposition process. As observed in Figure 6a,d,e, and Figure S18c,d (Supporting Information), both the Zn cross‐section and the surface remained flat, uniform, and regulated. That huge morphology difference suggested introducing TEGDME could effectively suppress Zn dendrites growth. Notably, the hybrid electrolyte enabled an ultralong lifetime of over 6000 h in a Zn symmetrical cell at 1 mA cm^−2^, ten times longer than the aqueous electrolyte (Figure 6f). A Zn‐Te_1_S_7_ pouch cell with a cathode size of 3 cm × 3 cm also showed an impressive areal capacity (3 mAh cm^−2^) using the same TEGDME‐added electrolyte (Figure 6g; Figure S19, Supporting Information). The cycling stability of the pouch cells could be further improved by applying external pressure to balance the volume change of the Te_1_S_7_/C cathode and Zn anode, conducting electrochemical activation at slow rates before cycling, and developing functional additives to modify the electrode surface.

What's more, the discharge plateau uplifted to 0.8 V with a higher areal capacity of 2.64 mAh cm^−2^ and smaller polarization of 0.62 V (vs. 2.02 mAh cm^−2^ and 0.70 V in aqueous electrolyte) with this TEGDME‐added electrolyte at the cut‐off voltage of 0.1–1.7 V (Figure 6h). In particular, our *operando* differential electrochemical mass spectrometry (DEMS) monitored the real‐time gas evolution of the Zn‐Te_1_S_7_/C battery in aqueous and hybrid electrolyte systems. As seen in Figure 6i, the battery with the electrolyte of 1 M ZnSO_4_ in H_2_O produced a high‐intensity H_2_ concentration during the charging process, as marked in grey color in Figure 6h, while the hybrid electrolyte induced a smaller amount of H_2_ when ZnS was converted back to S. The suppressed HER can be attributed to the decreased water activity by TEGDME.^[^ ^10^ ^]^ Besides, the TEGDME‐added electrolyte displayed a superior areal capacity over 50 cycles (500 h), but the Zn‐Te_1_S_7_/C battery with aqueous electrolyte only survived 16 cycles (160 h) followed by a sudden short circuit (Figure 6j). Therefore, employing organic TEGDME as a co‐solvent is a proven strategy to regulate Zn deposition morphology, inhibit Zn dendrites growth, suppress HER side reaction, and eventually extend the lifetime of the high‐capacity Zn‐Te_1_S_7_/C battery.

The volume ratio of H_2_O:TEGDME is critical in affecting the cycling performance of the Te_1_S_7_/C cathodes, as shown in Figure S20 (Supporting Information). Despite close initial specific capacities, the 9.5:0.5 volume ratio leads to a higher retained capacity (986.8 mAh g^−1^) after 25 cycles than the 9:1 ratio (865.2 mAh g^−1^) at the cut‐off voltage of 0.1–1.7 V under 0.2 A g^−1^. The inferior cycling stability could be attributed to the different numbers of water molecules in the Zn^2+^ solvation structure.^[^ ^16^ ^]^ Therefore, a proper addition of TEGDME co‐solvent is helpful in adjusting the Zn^2+^ solvation process and manipulating the Zn‐Te_1_S_7_ electrochemical conversion.

Besides, the S‐only cathode was also tested in the hybrid electrolyte (1 M ZnSO_4_ in H_2_O/TEGDME) under the same testing conditions for comparison. As illustrated in Figure S21 (Supporting Information), the 2nd‐cycle profile shows a primary flat discharge plateau at ≈0.75 V with a large polarization of 0.93 V. The Zn‐S/C battery in the TEGDME‐based electrolyte also demonstrated rapid capacity fading in the initial cycles (1682.5, 843.2, 192.5 mAh g^−1^ for the 1st, 2nd, and 5th cycle, respectively). In contrast, our Te‐doped S/C cathode possesses a smaller polarization of 0.62 V and retains 1274.7 mAh g^−1^ after 5 cycles under the same testing conditions (0.2 A g^−1^, 0.1–1.7 V) (Figure S20, Supporting Information).

The selection of co‐solvent was also carried out in this work, including acetonitrile (AN), dimethyl sulfoxide DMSO, and TEGDME. The volume ratio of H_2_O:AN or H_2_O:DMSO is fixed at 9.5:0.5 for a fair comparison with H_2_O/TEGDME. Similar to the H_2_O/TEGDME solvents, both batteries tested with AN and DMSO co‐solvents demonstrate one primary discharge plateau (Figures S22 and S23, Supporting Information), indicating a one‐step redox conversion from Te_1_S_7_ to ZnS/ZnTe. Unfortunately, the battery with DMSO retains inferior specific capacities in the 5th cycle (1103.7 for AN and 779.9 mAh g^−1^ for DMSO) than that with TEGDME (1199.2 mAh g^−1^) (Figure S20, Supporting Information) within the same voltage range of 0.1–1.7 V. Based on the above discussions, proper amount of suitable co‐solvent plays an important role in manipulating the Zn‐S conversion and maintaining good cycling stability of Zn‐Te_1_S_7_/C batteries.

The effect of electrolytes on the Te_1_S_7_/C cathode can also be analyzed from the perspective of the electrolyte‐cathode interface. It was revealed that organic solvents demonstrate stronger affinity to the S cathodes than water, resulting in better electrolyte wettability and faster charge transfer at interfaces.^[^ ^37^ ^]^ Specifically, the polar aprotic solvent TEGDME possesses more negative adsorption energies on the surfaces of S and ZnS (−0.71 and −2.71 eV, respectively) compared to free water (adsorption energies of −0.28 eV and −2.24 eV, respectively, on S and ZnS surfaces), suggesting better adherence to S or ZnS cathode surfaces.^[^ ^10^ ^]^ Besides, the organic cosolvent can reduce the coordination between Zn^2+^ and water molecules and suppress water decomposition due to the inhibited free water activity.^[^ ^37^, ^38^ ^]^ This improved wettability to the Te_1_S_7_/C cathode and mitigated side reaction together promoted the Zn^2+^ transfer in the Te_1_S_7_/C cathode and accelerated the redox kinetics, and finally contributed to a better Zn‐Te_1_S_7_/C battery with extended cycle life and smaller polarization.

The fast capacity fading in aqueous Zn‐Te_1_S_7_/C batteries (Figure 3g) is mainly caused by two factors. On the one hand, the electrochemical stability of Zn‐S is heavily affected by the aggressively formed Zn dendrites and hydrogen evolution side reaction (HER).^[^ ^13^, ^18^, ^39^ ^]^ The fast capacity fading is likely caused by the solvation effect of Zn^2+^ in aqueous electrolytes.^[^ ^13^ ^]^ The negative impact of Zn dendrites and HER is continuously accumulated over repeated cycling. Each discharge/charge cycle at 0.2 A g^−1^ takes 1.5 and 10.0 h (the average cycle duration calculated from the initial 10 cycles) for Zn‐Te_1_S_7_/C and Zn‐S/C batteries, respectively. It is speculated that the cycling instability is easier to observe for the longer‐calendar‐life Zn‐Te_1_S_7_/C battery, compared to the Zn‐S/C battery. This speculation is supported by our mechanism analysis through in‐situ OM and DEMS techniques in Figure 6. Introducing the TEGDME co‐solvent can regulate Zn dendrites growth and inhibit HER side reaction by reducing the number of water molecules in Zn^2+^ and suppressing the solvation effect of Zn^2+^, thus contributing to an extended cycle number, life duration (497 h) and capacity retention (91.3%, for the initial 10 cycles) of the Zn‐Te_1_S_7_/C battery. In contrast, the Zn‐Te_1_S_7_/C battery experiences a sudden short circuit after only 161 h (retained 75.2% after 10 cycles) under the same current density of 0.2 A g^−1^ and a cut‐off voltage of 0.1–1.7 V. This improved capacity retention is indirect evidence to explain the capacity degradation phenomenon in the Zn‐Te_1_S_7_/C battery. On the other hand, the rapid capacity fading could originate from the shuttle effect caused by the dissolution of polysulfides or polytellurides intermediates into the aqueous solution.^[^ ^19^, ^26^ ^]^ The multi‐step discharge process (S_8_ → S_x_^2−^ → S^2−^ or Te → Te_x_^2−^ → Te^2−^) ^[^ ^16^, ^26^ ^]^ is also observed from the two distinct discharge plateaus in Figure 3d. By employing the TEGDME‐adjusted hybrid electrolyte, the two discharge plateaus have merged into one for the Zn‐Te_1_S_7_/C battery (Figure 6h), indicating the one‐step solid‐state conversion (Te_1_S_7_ + 8Zn^2+^ + 16e^−^ ↔ 7ZnS + ZnTe). Our conclusion is that the durable and high‐capacity Zn‐Te_1_S_7_ battery requires both materials' design from the Zn anode and Te_1_S_7_ cathode. Moving forward, our future plans involve introducing functional additives such as I_2_ or incorporating an artificial interlayer on the cathode or separator surface. These measures aim to further enhance the interaction between the electrolyte and electrode in the Zn‐Te_1_S_7_ battery system, addressing the observed challenges and potentially improving overall performance.

## 3 Conclusions

In summary, this work demonstrates a novel high‐areal‐capacity conversion‐type cathode Te_1_S_7_/C for aqueous Zn batteries. Te's excellent electric conductivity significantly contributes to reshaping the Zn‐S electrochemistry and enhancing the battery's reversible specific or areal capacities. Kinetics analysis reveals that the incorporation of Te in the S cathode reduces the reaction polarization, elevates the discharge plateau, and facilitates the reversible S‐ZnS conversion. Moreover, the small amount of Te can be transformed into ZnTe to fulfill its unique redox reaction while S is converted to ZnS, as investigated by ex situ HRTEM, SAED, and XPS analysis. These advantages of Te make Te_1_S_7_/C an outstanding cathode to compete with other S cathodes or intercalation‐type cathodes. Specifically, the Zn‐Te_1_S_7_/C battery showcases an impressive reversible capacity of 1335.0 mAh g^−1^ at 0.1 A g^−1^ and realizes a remarkable areal capacity of 5.64 mAh cm^−2^ with the TeS mass loading of 4.22 mg cm^−2^. More importantly, our hybrid electrolyte can suppress Zn dendrites growth and extend the lifetime of Zn‐Te_1_S_7_ batteries, as investigated by in‐situ OM and DEMS techniques. This proposed cathode design offers a reliable strategy to boost Zn‐S chemistry and achieve high energy density for energy storage devices.

## 4 Experimental Section

### Chemicals

The chemicals used in this work include tellurium powder (Fenix Advanced Materials, 99.99%), sulfur powder (Alfa Aesar, 100 mesh, 99.5%), Ketjen black (KB, EC600), zinc sulfate (ZnSO_4_, Ward's Science), tetraethylene glycol dimethyl ether (TEGDME, Sigma–Aldrich), polytetrafluoroethylene (PTFE, MSE supplies, 1.5 g cm^−3^) Acetonitrile (AN, Sigma–Aldrich), Dimethyl sulfoxide (DMSO, Sigma–Aldrich), polyvinylidene fluoride (PVDF, MTI), and N‐Methyl‐2‐pyrrolidone (NMP, Alfa Aesar). All the chemicals were used as received without further purification.

### Synthesis of Te_1_S_7_ and Te_1_S_7_/C

The mixture of Te and S powders with a Te/S mole ratio of 1:7 was first ball‐milled and then sealed in a quartz tube under vacuum. The mixture was subsequently heated in a tube furnace at 450 °C for 10 h, followed by a natural cooling down process to room temperature. The as‐obtained Te‐S solid solution was denoted as the Te_1_S_7_ compound. The synthesis of Te_2_S_6_, Te_3_S_5_, Te_4_S_4_, Te_5_S_3_, Te_6_S_2_, and Te_7_S_1_ (Te/S mole mixing ratio is 2:6, 3:5, 4:4, 5:3, 6:2, 7:1, respectively) followed the same preparation procedures.

The Te_1_S_7_/C composite was prepared by a melt diffusion method. A powder mixture of Te_1_S_7_ and KB with a mass ratio of 6:4 was ball‐milled and sealed in a quartz tube under vacuum. Afterward, the mixture was heated at 480 °C for 10 h for full TeS infusion into carbon pores. After the temperature cooled down to room temperature, the as‐prepared powder product was denoted as Te_1_S_7_/C. The S/C and Te/C composites were also prepared by heating S/C and Te/C mixtures (the mass ratio was also 6:4) at 160 °C in an autoclave (for S/C) and 480 °C for 10 h in a tube furnace under a nitrogen atmosphere (for Te/C).

### Preparation of Free‐Standing Te_1_S_7_/C Cathodes

The as‐prepared Te_1_S_7_/C composite was mixed with super P and PTFE at the mass ratio of 7:2:1 in isopropyl alcohol solvent to form a uniform slurry. Then the slurry was ground and rolled into a dough, which was dried at 60 °C overnight for residual solvent evaporation. The dried slurry was then cut into circular cathodes with a 12 mm diameter for cell assembly. The areal mass loading of Te_1_S_7_ in the Te_1_S_7_/C cathode in this work was 1–6 mg cm^−2^.

For comparison, the conventional Te_1_S_7_/C cathode was also prepared by mixing Te_1_S_7_/C, super P, and PVDF (2 wt.% in NMP). The slurry was cast onto a piece of Ti foil, dried at 60 °C overnight, and cut into 12 mm‐diameter pellets for cell assembly.

### Morphology and Structure Observations

Scanning electron morphology (SEM) and high‐resolution transmission electron microscopy (HRTEM) were used to create images of the morphologies of powder and electrodes in this work. The elemental composition information of materials was collected by energy dispersive spectroscopy (EDS). X‐ray diffraction (XRD) patterns of samples (powder, electrodes at different states of charge/discharge) were measured for phase identification using Bruker D8‐Advance X‐ray diffractometer under 40 kV and 40 mA. The chemical states of charged/discharged electrodes were characterized by X‐ray photoelectron spectroscopy (XPS). Thermogravimetric analysis (TGA) of Te_1_S_7_/C and S/C was carried out in a nitrogen atmosphere.

### Electrochemical Characterizations

The 2032‐type Zn‐S or Zn‐Te_1_S_7_ coin cells were assembled in the air. The Te_1_S_7_/C (S/C), Zn foil, and Whatman Glass Fiber (GF/F) were used as the cathode, anode, and separator, respectively. Each coin cell consumed 160 µL electrolyte (1 M ZnSO_4_ in H_2_O or 1 M ZnSO_4_ in H_2_O/TEGDME (volume ratio 9.5:0.5)). The electrolytes of 1 M ZnSO_4_ in H_2_O/AN (volume ratio 9.5:0.5) and 1 M ZnSO_4_ in H_2_O/DMSO (volume ratio 9.5:0.5) were also prepared and tested for comparison. Electrochemical impedance spectroscopy (EIS) measurement was carried out on EC‐lab with 5 mV AC amplitude and frequency range from 1 MHz to 1 Hz. Cyclic voltammogram (CV) measurements were performed at scan rates of 0.2 mV s^−1^. Galvanostatic measurements were tested at room temperature at 0.1–1.5 V or 0.1–1.7 V using a NEWARE battery cycler (CT‐4008T‐5V50mA‐164, Shenzhen, China). The specific capacity of the battery is calculated based on the mass of active materials (Te_1_S_7_ or S). The galvanostatic intermittent titration technique (GITT) test was performed by applying a constant current density of 50 mA g^−1^ to the Zn‐Te_1_S_7_ (Zn‐S) cell for 1 h and leaving the cell rest for 4 h until the charge/discharge cut‐off voltage limit. To identify the charge/discharge products, the Zn‐S and Zn‐Te_1_S_7_ cells were conducted for 2 full cycles, and then were discharged to 0.1 V, or charged to 1.5 V at the current density of 0.2 A g^−1^. Later, these cells were disassembled, and the cathodes were rinsed with H_2_O and naturally dried for further SEM, TEM, and XPS analysis.

In‐situ optical microscopy (OM) was used to capture dynamic Zn plating/stripping behaviors while the symmetrical Zn battery with aqueous or hybrid electrolyte was under charge/charge conditions at a current density of 2 mA cm^−2^ for 1 h. After 5 cycles, all Zn foils were collected and characterized by SEM.

Differential electrochemical mass spectrometry (DEMS) measurement was performed on an HPR‐40 instrument (Hiden Analytical Ltd.) to analyze the in‐situ gas evolution during discharge/charge process in a Zn‐Te_1_S_7_/C battery. The sealed testing cell is comprised of Zn anode (100 µm thickness), Te_1_S_7_/C cathode (2.6 mg cm^−2^), glass fiber separator (GF/F, Whatman), 160 µL electrolyte (1 M ZnSO_4_ in H_2_O or 1 M ZnSO_4_ in H_2_O/TEGDME) and stainless‐steel spacer (1 mm). Before testing, the system was deflated with helium gas (purity 99.999%) for 24 h under a flow rate of 5 mL min^−1^) until an ultra‐high vacuum was reached. While the DEMS cell was discharged/charged by NEWARE Battery Tester, the resulting gas species (m/z = 2 for H_2_) was collected and analyzed.

For pouch cell assembly, the Te_1_S_7_/C cathode sheet with a mass loading of 2.6 mg cm^−2^ was cut into 3 cm × 3 cm and attached to a piece of Ti foil. Zn anode was prepared by cutting a 100 µm thick Zn sheet into 3 cm x 3 cm. Subsequently, Zn anode, glass fiber, and Te_1_S_7_/C cathode were stacked and placed inside the aluminum‐laminated case. 800 uL hybrid electrolyte of 1 M ZnSO_4_ in H_2_O/TEGDME was injected into the battery pack, which was further sealed by MSK‐115A‐S hot sealing machine. Finally, the Zn‐Te_1_S_7_ pouch cell was connected to the NEWARE workstation for cycling tests.

## Conflict of Interest

The authors declare no conflict of interest.

## Supporting information

Supporting Information

## Data Availability

The data that support the findings of this study are available from the corresponding author upon reasonable request.

## References

[1] a) Z. Ye , Z. Cao , M. O. Lam Chee , P. Dong , P. M. Ajayan , J. Shen , M. Ye , Energy Storage Mater. 2020, 32, 290;

[2] a) P. Cai , K. Wang , J. Ning , X. He , M. Chen , Q. Li , H. Li , M. Zhou , W. Wang , K. Jiang , Adv. Energy Mater. 2022, 12, 2202182;

[3] a) M. Yan , P. He , Y. Chen , S. Wang , Q. Wei , K. Zhao , X. Xu , Q. An , Y. Shuang , Y. Shao , K. T. Mueller , L. Mai , J. Liu , J. Yang , Adv. Mater. 2018, 30; 10.1002/adma.201703725 29131432

[4] a) D. Wang , L. Wang , G. Liang , H. Li , Z. Liu , Z. Tang , J. Liang , C. Zhi , ACS Nano 2019, 13, 10643; 31419380 10.1021/acsnano.9b04916

[5] a) G. Zampardi , F. L. Mantia , Curr. Opin. Electrochem. 2020, 21, 84;

[6] a) L. E. Blanc , D. Kundu , L. F. Nazar , Joule 2020, 4, 771;

[7] N. Zhang , X. Chen , M. Yu , Z. Niu , F. Cheng , J. Chen , Chem. Soc. Rev. 2020, 49, 4203. 32478772 10.1039/c9cs00349e

[8] L. W. Luo , C. Zhang , X. Wu , C. Han , Y. Xu , X. Ji , J. X. Jiang , Chem Commun (Camb) 2021, 57, 9918. 34498654 10.1039/d1cc04337d

[9] a) G. Chang , J. Liu , Y. Hao , C. Huang , Y. Yang , Y. Qian , X. Chen , Q. Tang , A. Hu , Chem. Eng. J. 2023, 457, 141083;

[10] M. Yang , Z. Yan , J. Xiao , W. Xin , L. Zhang , H. Peng , Y. Geng , J. Li , Y. Wang , L. Liu , Z. Zhu , Angew. Chem. Int. Ed. Engl. 2022, 61, e202212666. 36056534 10.1002/anie.202212666

[11] J. Chen , W. Zhao , J. Jiang , X. Zhao , S. Zheng , Z. Pan , X. Yang , Energy Storage Mater. 2023, 59, 102767.

[12] Z. Liu , L. Li , L. Qin , S. Guo , G. Fang , Z. Luo , S. Liang , Adv. Mater. 2022, 34, 2204681. 10.1002/adma.202204681 35951631

[13] Z. Xu , Y. Zhang , W. Gou , M. Liu , Y. Sun , X. Han , W. Sun , C. Li , Chem Commun (Camb) 2022, 58, 8145. 35775961 10.1039/d2cc02075k

[14] T. Zhou , H. Wan , M. Liu , Q. Wu , Z. Fan, Y. Zhu, Mater. Today Energy 2022, 27, 101025.

[15] W. Li , K. Wang , K. Jiang , Adv. Sci. 2020, 7, 2000761. 10.1002/advs.202000761 PMC7709974 33304742

[16] Y. Guo , R. Chua , Y. Chen , Y. Cai , E. J. J. Tang , J. J. N. Lim , T. H. Tran , V. Verma , M. W. Wong , M. Srinivasan , Small 2023, 19, e2207133. 36971296 10.1002/smll.202207133

[17] M. Cui , J. Fei , F. Mo , H. Lei , Y. Huang , ACS Appl. Mater. Interfaces 2021, 13, 54981. 34780154 10.1021/acsami.1c15750

[18] Y. Zhao , D. Wang , X. Li , Q. Yang , Y. Guo , F. Mo , Q. Li , C. Peng , H. Li , C. Zhi , Adv. Mater. 2020, 32, 2003070. 10.1002/adma.202003070 32596928

[19] W. Zhang , M. Wang , J. Ma , H. Zhang , L. Fu , B. Song , S. Lu , K. Lu , Adv. Funct. Mater. 2023, 33, 2210899.

[20] H. Zhang , Z. Shang , G. Luo , S. Jiao , R. Cao , Q. Chen , K. Lu , ACS Nano 2022, 16, 7344. 34889091 10.1021/acsnano.1c08645

[21] a) Z. Chen , F. Mo , T. Wang , Q. Yang , Z. Huang , D. Wang , G. Liang , A. Chen , Q. Li , Y. Guo , X. Li , J. Fan , C. Zhi , Energy Environ. Sci. 2021, 14, 2441;

[22] W. Li , Y. Ma , P. Li , X. Jing , K. Jiang , D. Wang , Adv. Funct. Mater. 2021, 31, 2101237.

[23] J. Kang , Z. Zhao , H. Li , Y. Meng , B. Hu , H. Lu , Energy Mater 2022, 2, 200009.

[24] Y. Zhang , W. Lu , P. Zhao , M. H. Aboonasr Shiraz , D. Manaig , D. J. Freschi , Y. Liu , J. Liu , Carbon 2021, 173, 11.

[25] a) S. Li , J. Ma , Z. Zeng , W. Hu , W. Zhang , S. Cheng , J. Xie , J. Mater. Chem. 2020, 8, 3405;

[26] a) Z. Chen , Q. Yang , F. Mo , N. Li , G. Liang , X. Li , Z. Huang , D. Wang , W. Huang , J. Fan , C. Zhi , Adv. Mater. 2020, 32, 2001469; 10.1002/adma.202001469 32924220

[27] a) Y. Lu , C.‐Z. Zhao , H. Yuan , J.‐K. Hu , J.‐Q. Huang , Q. Zhang , Matter 2022, 5, 876;

[28] R. Tao , B. Steinhoff , C. H. Sawicki , J. Sharma , K. Sardo , A. Bishtawi , T. Gibbs , J. Li , J. Power Sources 2023, 580, 233379.

[29] a) W. Yao , M. Chouchane , W.‐K. Li , S. Bai , Z. Liu , L. Li , A. X. Chen , B. Sayahpour , R. Shimizu , G. Raghavendran , M. Schroeder , Y.‐T. Chen , D. H. S. Tan , B. Sreenarayanan , C. K. Waters , A. Sichler , B. Gould , D. J. Kountz , D. J. Lipomi , M. Zhang , Y. S. Meng , Energy Environ. Sci. 2023, 16;

[30] X. Ji , K. T. Lee , L. F. Nazar , Nat. Mater. 2009, 8, 500. 19448613 10.1038/nmat2460

[31] a) Y. Li , M.‐Q. Wang , Y. Chen , L. Hu , T. Liu , S. Bao , M. Xu , Energy Storage Mater. 2018, 10, 10;

[32] J. Zhang , Y.‐X. Yin , Y. You , Y. Yan , Y.‐G. Guo , Energy Technol. 2014, 2, 757.

[33] a) C. Jones , M. Mauguin , J. Chem. Phys. 1977, 67, 1587;

[34] a) L. Zhou , Y. Zheng , M. Ouyang , L. Lu , J. Power Sources 2017, 364, 242;

[35] J. Huang , X. Xie , K. Liu , S. Liang , G. Fang , Energy Environ. Mater. 2022, 6.

[36] a) J. Li , Z. Cheng , Z. Li , Y. Huang , Mater. Horiz. 2023, 10, 2436; 37140157 10.1039/d3mh00278k

[37] a) D. Wang , Q. Li , Y. Zhao , H. Hong , H. Li , Z. Huang , G. Liang , Q. Yang , C. Zhi , Adv. Energy Mater. 2022, 12, 2102707;

[38] a) T. C. Li , Y. Lim , X. L. Li , S. Luo , C. Lin , D. Fang , S. Xia , Y. Wang , H. Y. Yang , Adv. Energy Mater. 2022, 12, 2103231;

[39] X. Wang , L. Liu , Z. Hu , C. Peng , C. Han , W. Li , Adv. Energy Mater. 2023, 13, 2302927.

