# Supplementary material for: A Tellurium‐Boosted High‐Areal‐Capacity Zinc‐Sulfur Battery
Source: Adv Sci (Weinh). 2024 Apr 2;11(23):2308580. doi: 10.1002/advs.202308580 (PMC11187902; doi:10.1002/advs.202308580)
Supplement: Supplementary file 1 — Supporting Information [file ADVS-11-2308580-s001.pdf]

ADVANCED SCIENCE Open Access

Supporting Information

for *Adv. Sci.*, DOI 10.1002/advs.202308580

A Tellurium-Boosted High-Areal-Capacity Zinc-Sulfur Battery

*Yue Zhang, Amardeep Amardeep, Zhenrui Wu, Li Tao, Jia Xu, Donald J. Freschi and Jian Liu**

A Tellurium-Boosted High-Areal-Capacity Zinc-Sulfur Battery

Yue Zhang,[a,b] Amardeep Amardeep,[a] Zhenrui Wu,[a] Li Tao,[a] Jia Xu,[a] Donald J. Freschi,[c] Jian Liu,[a,b,*]

[a] *School of Engineering, Faculty of Applied Science, University of British Columbia, Kelowna, BC V1V 1V7, Canada*

[b] *Pacific Institute for Climate Solutions and School of Environmental Studies, University of British Columbia, Kelowna, BC V1V 1V7, Canada*

[c] *Fenix Advanced Materials, 2950 Highway Drive, Trail BC, V1R 2T3, Canada*

[*] *Corresponding authors' email: Jian.liu@ubc.ca (J. Liu)*

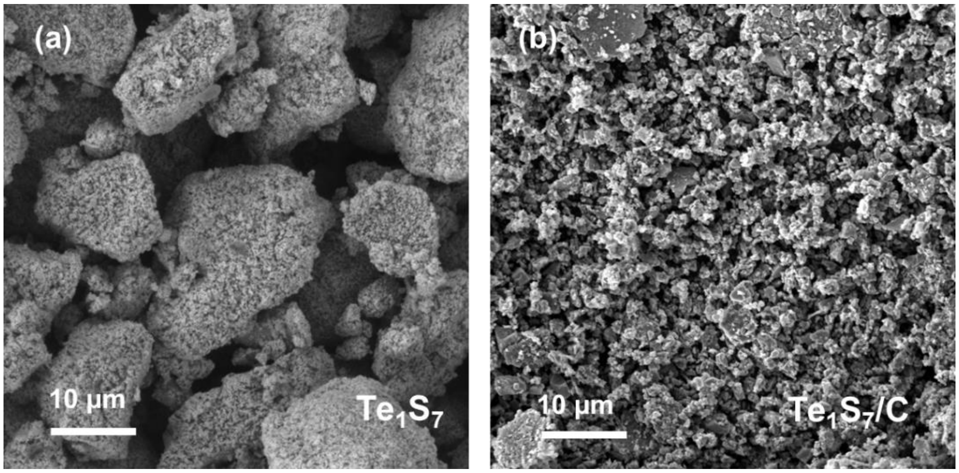

Figure S1. SEM images of (a) Te_1S_7 and (b) Te_1S_7/C particles.

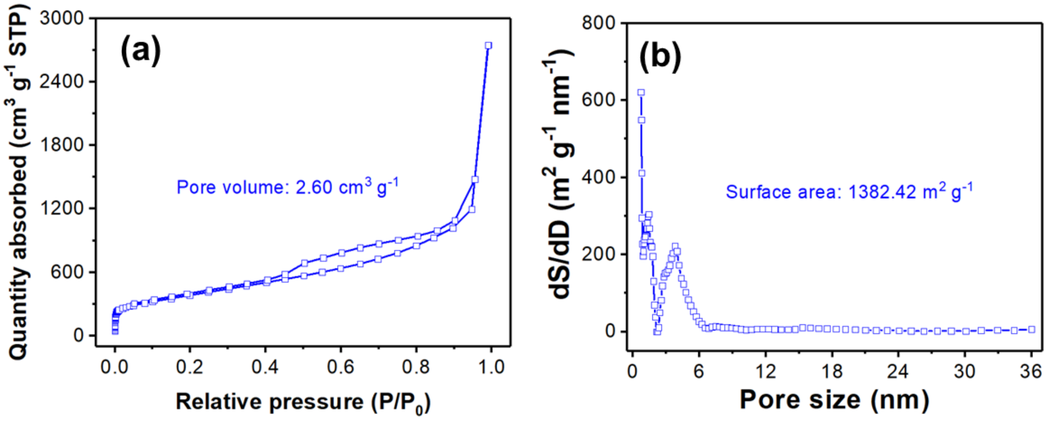

Figure S2. (a) Nitrogen adsorption/desorption isotherm of KB, and (b) pore size distribution of KB host.

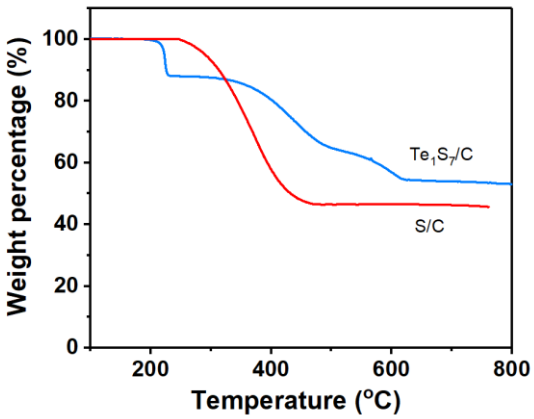

Figure S3. TGA curves of Te_1S_7/C and S/C composites.

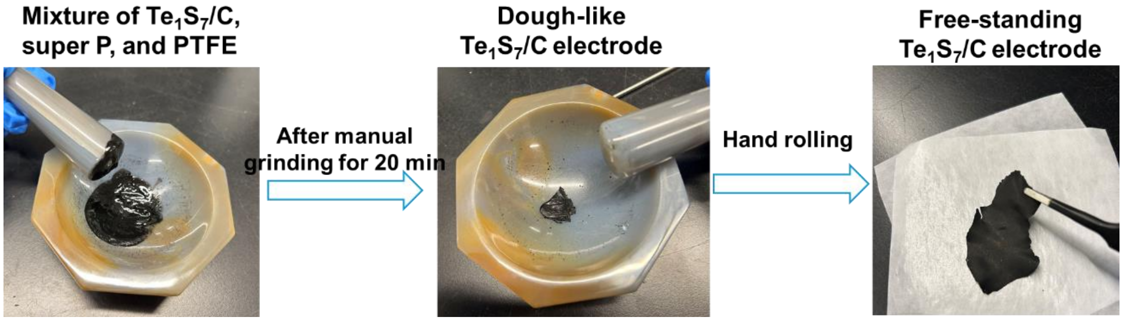

Figure S4. Preparation procedures of the free-standing Te_1S_7/C electrodes.

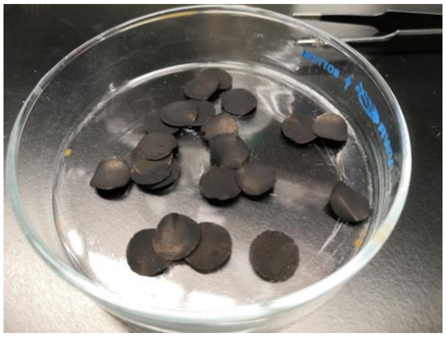

Figure S5. Digital photo of free-standing Te_1S_7/C cathodes.

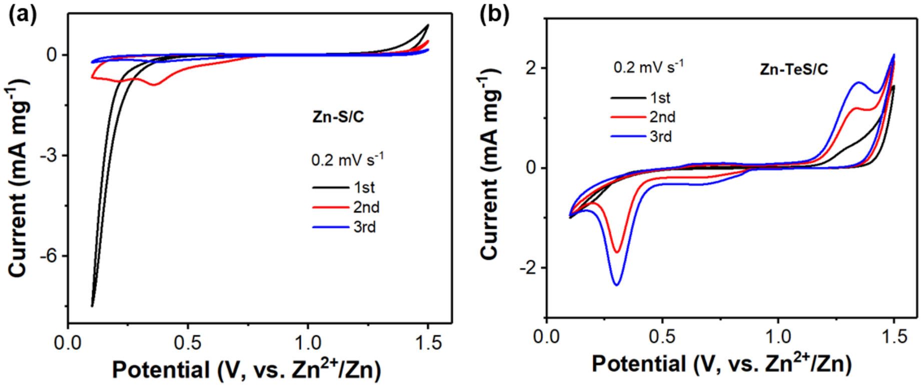

Figure S6. CV plots of (a) Zn-S/C and (b) Zn-Te_1S_7/C batteries at the scan rate of 0.2 mV s^{-1}.

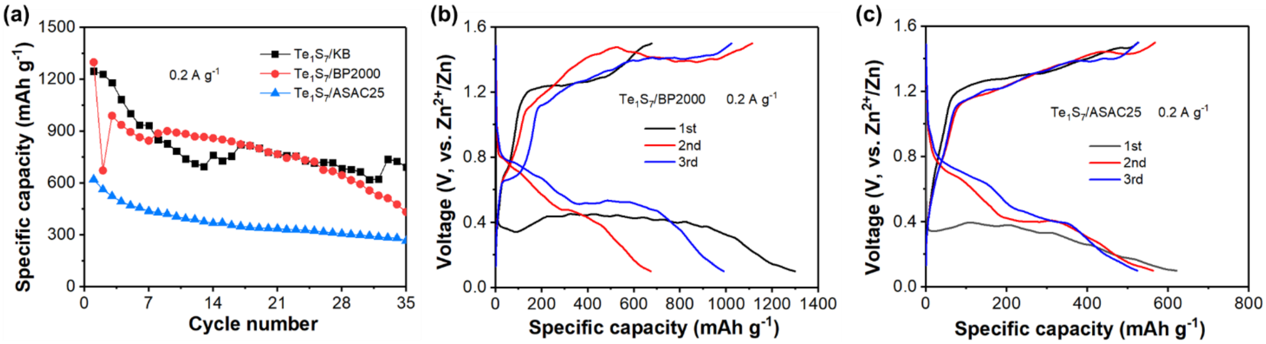

Figure S7. (a) Cycling performance of Te_1S_7 confined into different carbons (KB, BP2000, and ASAC25) at 0.2 A g^{-1}, the charge/discharge profiles of (b) Te_1S_7/BP2000, and (c) Te_1S_7/ASAC25 in the initial 3 cycles.

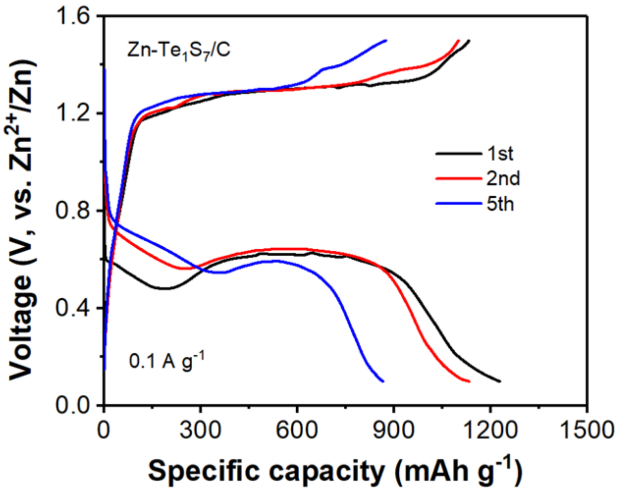

Figure S8. Galvanostatic charge/discharge profiles of the Te_1S_7/C cathode in the electrolyte of 1M $Zn(OTf)_2$ in H_2O.

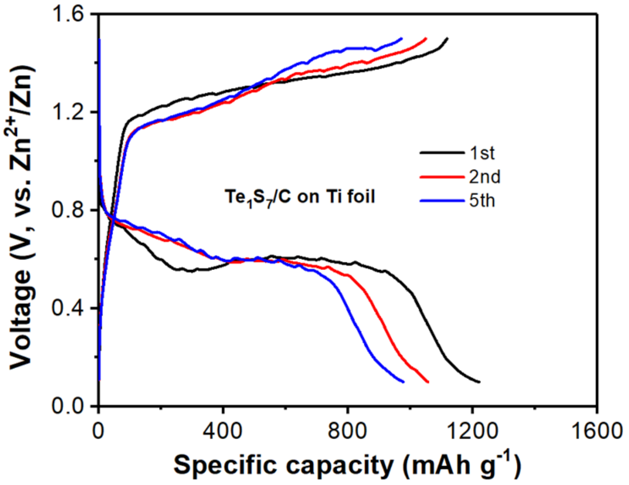

Figure S9. Galvanostatic charge/discharge profiles of the Te_1S_7/C cathode cast onto Ti foil with the mass loading of 0.6 mg cm^{-2} tested at 0.1 A g^{-1}.

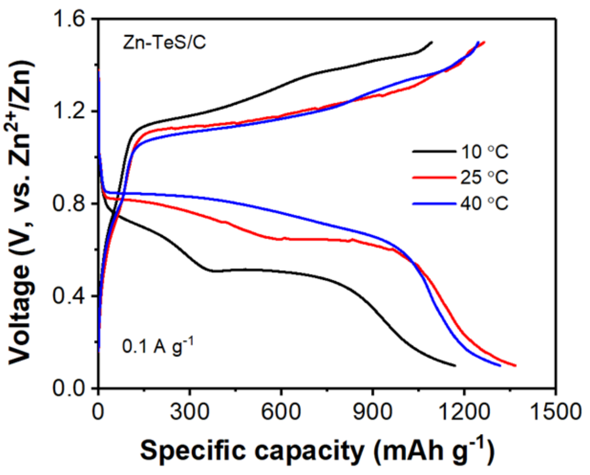

Figure S10. Galvanostatic charge/discharge profiles of the Zn-Te_1S_7/C battery with working temperatures of 10 °C, 25 °C, and 40 °C.

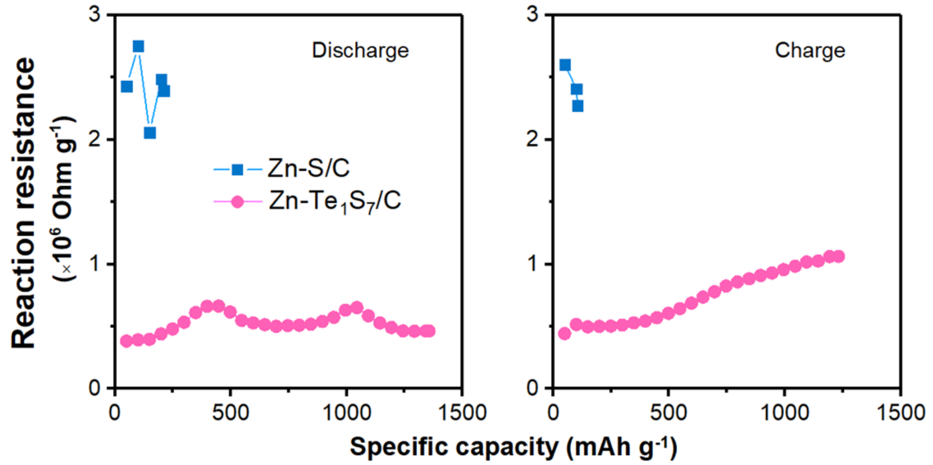

Figure S11. Reaction resistance of Zn-S/C and Zn-Te_1S_7/C batteries from GITT plots in Figure 3e and 3f during discharge and charge processes.

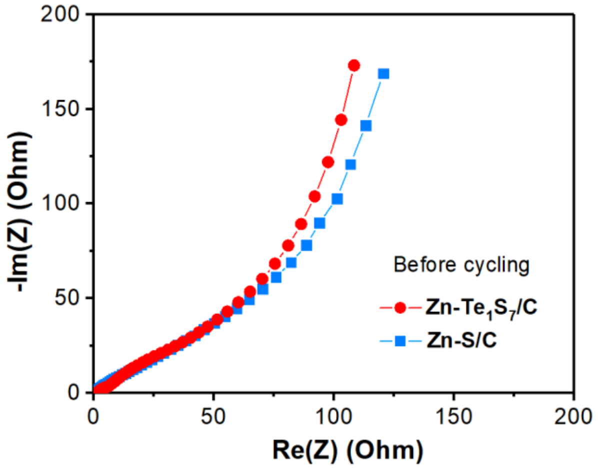

Figure S12. Nyquist plots of Zn-S/C and Zn-Te_1S_7/C batteries before cycling.

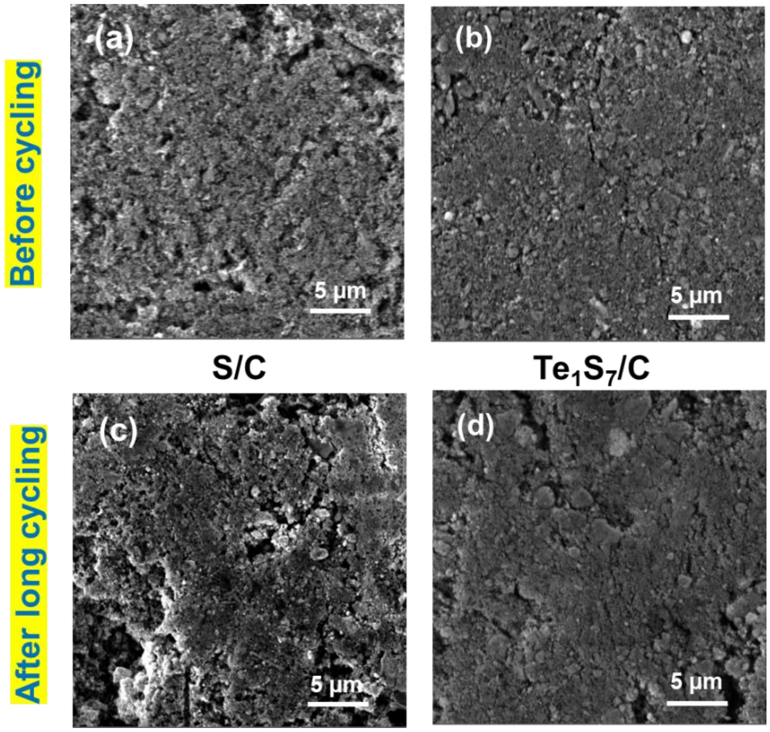

Figure S13. SEM images of pristine (a) S/C and (b) Te_1S_7/C cathodes, cycled (c) S/C and (d) Te_1S_7/C cathodes (after 5 cycles at 0.2 A g^{-1}).

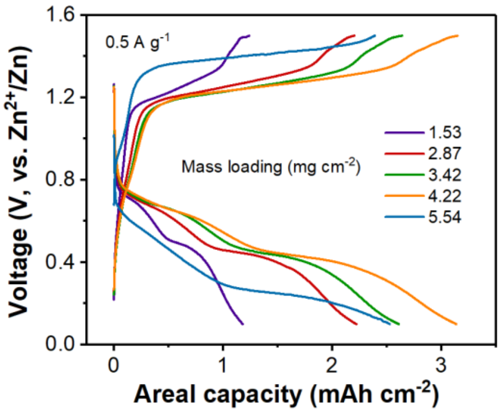

Figure S14. Galvanostatic charge/discharge profiles of the Zn-Te_1S_7/C battery with various mass loadings at 0.5 A g^{-1}.

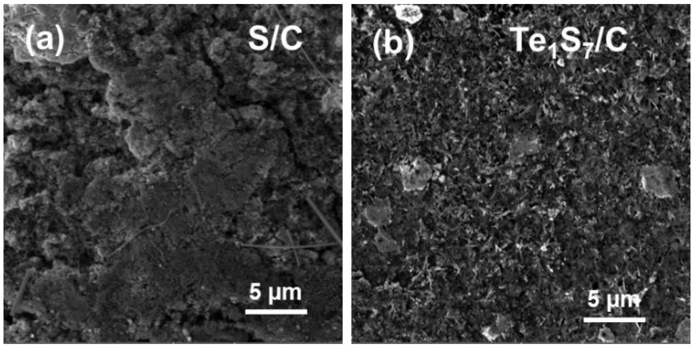

Figure S15. SEM images of fully discharged (a) S/C and (b) Te_1S_7/C cathodes.

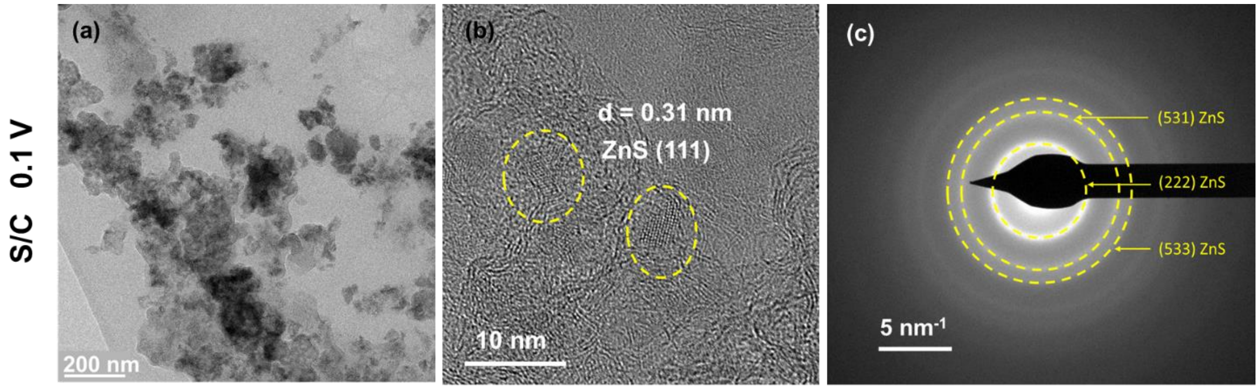

Figure S16. (a) TEM, (b) HRTEM, and (c) SAED images of the S/C electrode after being discharged to 0.1 V.

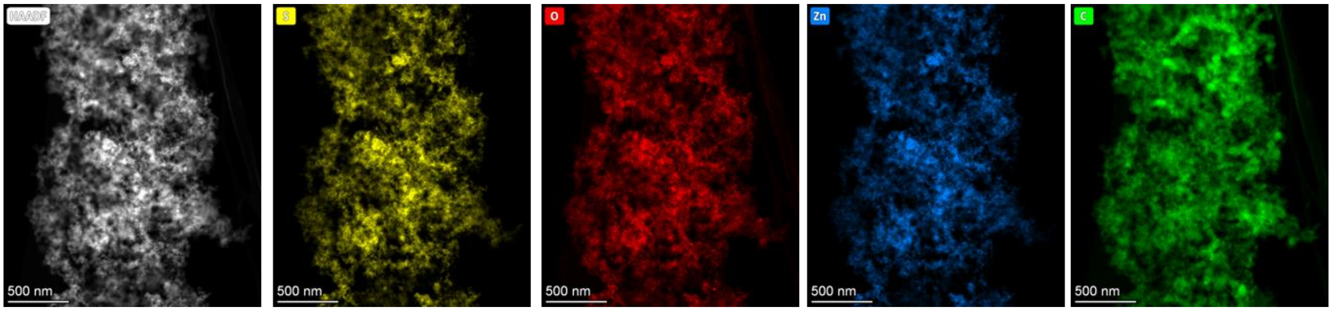

Figure S17. HADDF image and elemental mapping of the discharged S/C cathode.

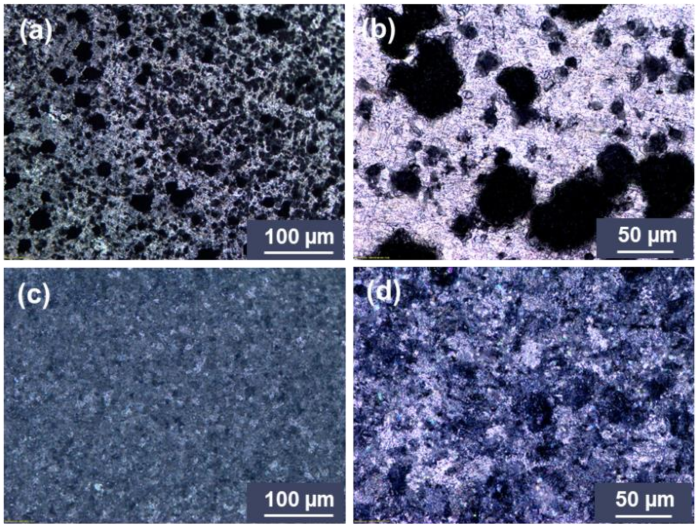

Figure S18. Optical microscopy images of Zn surface after 5 cycles of Zn plating/stripping in (a, b) aqueous and (c, d) hybrid electrolytes.

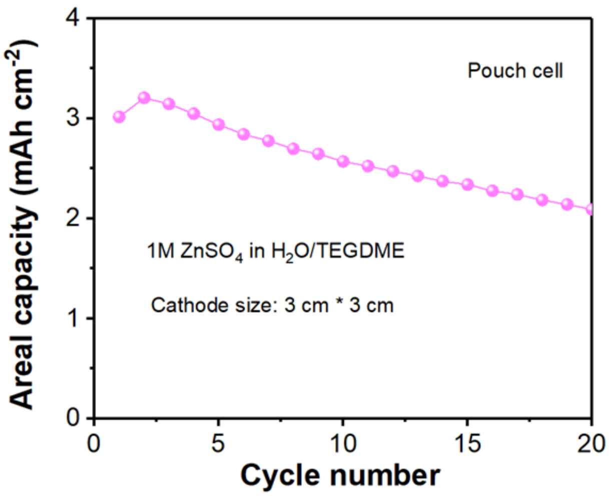

Figure S19. Areal capacities of the Zn-Te_1S_7 battery over 20 cycles with a hybrid electrolyte tested at 0.2 A g^{-1} with a cut-off voltage range of 0.1-1.7 V.

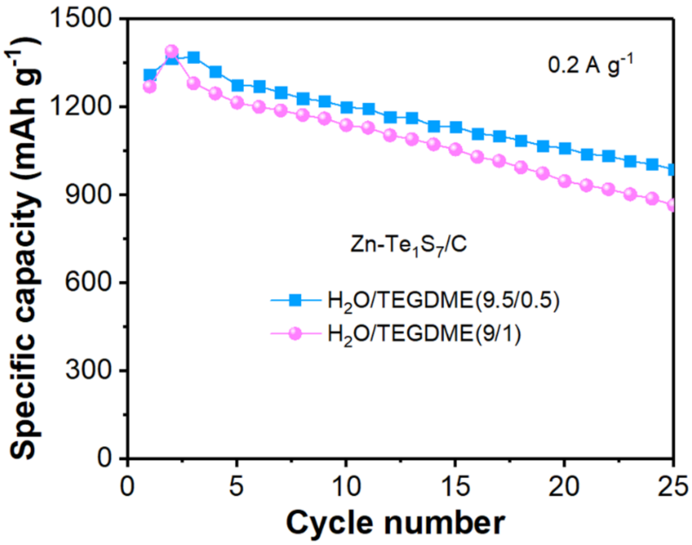

Figure S20. Specific discharge capacities of the Zn-Te_1S_7 battery over 25 cycles in the hybrid electrolyte with H_2O/TEGDME volume ratios of 9.5:0.5 and 9:1, tested at 0.2 A g^{-1} with a cut-off voltage range of 0.1-1.7 V.

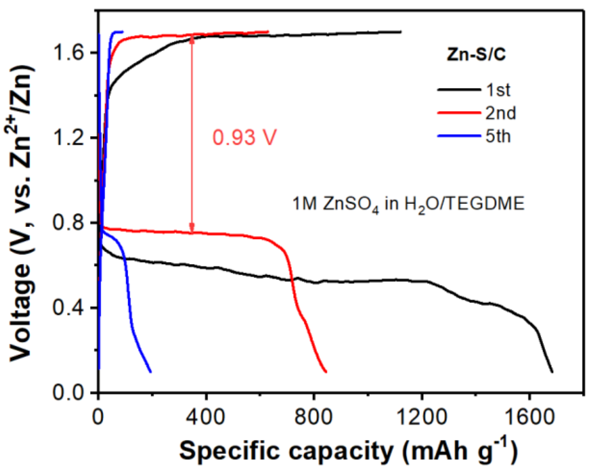

Figure S21. Galvanostatic charge/discharge profiles of the Zn-S/C battery in the hybrid electrolyte (1M $ZnSO_4$ in H_2O/TEGDME).

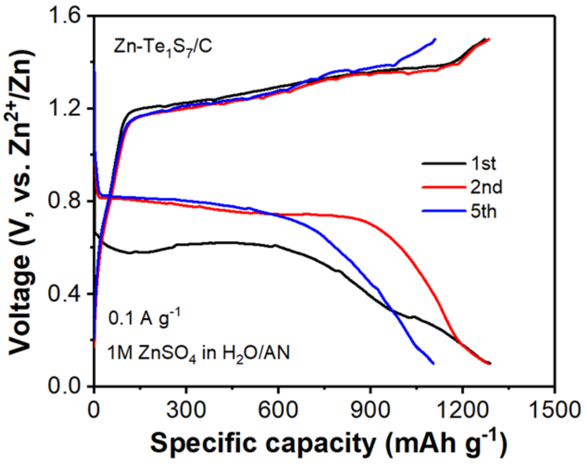

Figure S22. Galvanostatic charge/discharge profiles of the Te_1S_7/C cathode in the electrolyte of 1M $ZnSO_4$ in H_2O/AN (volume ratio: 9.5:0.5).

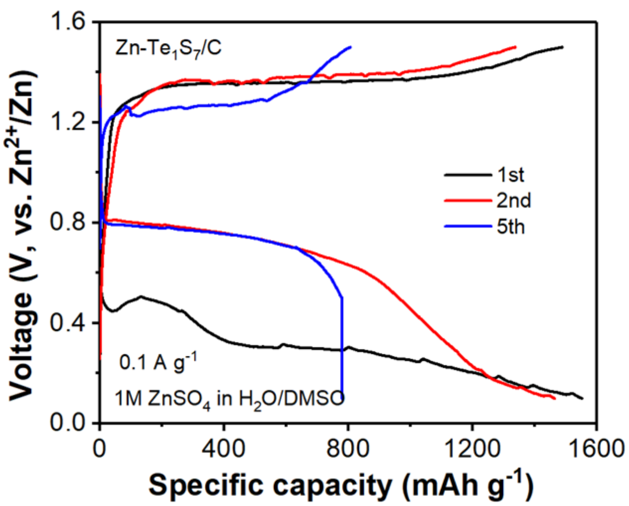

Figure S23. Galvanostatic charge/discharge profiles of the Te_1S_7/C cathode in the electrolyte of 1M $ZnSO_4$ in H_2O/DMSO (volume ratio: 9.5:0.5).

Table S1. Cathode comparison in aqueous Zn-ion batteries.

**Cathode name**	**Mass loading (mg cm^{-2})**	**Specific capacity (mAh g^{-1})**	**Highest areal capacity (mAh cm^{-2})**	**Discharge plateau (V)**	**Energy density (Wh kg^{-1})**	**Current density (A g^{-1})**	**Electrolyte**	**Ref.**
**Te_1S_7/C**	1.53-5.54	1335 (aqueous) 1365 (hybrid)	5.64 (aqueous)	0.7 (aqueous) 0.8 (hybrid)	934.6	0.1	1M $ZnSO_4$ in H_2O 1M $ZnSO_4$ in H_2O/TEGDME	This work
**S@CNTs-50**	0.7-3.04	1105	3.31*	0.5	502	0.1	1M $Zn(CH_3COO)_2$ + I_2	[1]
**LF-PLSD**	4	1148	4.59*	0.8	724.7	0.3	Hydrogel electrolyte with 1M $Zn(TFSI)_2$	[2]
**S@C**	1.0-1.5	846	1.27*	0.4	258.62	0.5	Zn salts + ChCl/urea	[3]
**KB-S (primary battery)**	8.3	1668	11.4	0.7	1083.3	0.05	1M $ZnCl_2$	[4]

**CNF-S**	Not given	667	Not given	0.3	283	0.5	$Zn(CH_3COO)_2$ + EG/I_2	[5]
**ZnS@CF**	1.5-3	465	1.4*	0.5	274	0.1	3M $ZnSO_4$ + TUI	[6]
**CMK-3@S**	1-2	788	1.58*	0.26	Not given	0.2	3M $Zn(OTf)_2$	[7]
**HCS/S**	1.2-1.6	1140	1.82*	0.4	Not given	0.5	2M $Zn(OTf)_2$ +G_4/I_2	[8]
**S@Fe-PANi**	1.8	1205	2.17*	0.6	720	0.2	2M $ZnSO_4$	[9]
**S@CNTs**	1.5-2.5	1116	2.79*	0.47	Not given	0.1	1M $Zn(CH_3COO)_2$ + PEG	[10]
**AC-S**	2.6	200	0.52*	0.3	Not given	1	ZnAc/ZnBF–I_2/EG	[11]
**S@KB**	1	1785.1	1.78*	0.45	803.3	1	2M $ZnSO_4$ + TU	[12]
**S@NPC**	3	1435	4.31*	0.5	730	0.1	$Zn(CF_3SO_3)_2$/ ZnI_2 + EG	[13]
**CMK-3@S**	2.0-2.5	1630	4	0.6	Not given	0.1	2M $ZnSO_4$ + ZnI_2	[14]
**S@FeNC/NC/CC**	2.0-2.6	1143	2.97*	0.61	Not given	0.2	2M $ZnSO_4$	[15]
**Te-MoS_2@NFC**	1.0-1.4	483	0.68*	0.3	172.4	0.15	3M $Zn(OTf)_2$	[16]
**Te-NSs**	1.5	419	0.63*	0.6	74.1	0.05	1M $ZnSO_4$	[17]
**TeO_2**	1	460	0.46*	0.6	Not given	0.05	1M $ZnSO_4$	[18]
**Te photocathode**	1.0-1.3	362	0.47*	0.6	Not given	0.1	3M $ZnSO_4$	[19]
**Te-MoO_2/NC**	1.0-1.5	493	0.74*	0.3	159.9	0.1	3M $Zn(OTf)_2$	[20]
**Te@CSs**	1.5, 14	650	7.13	0.8	542	0.1	30m $ZnCl_2$	[21]
**$MoTe_{1.7}$**	5	338	1.69*	0.2	137	0.2	3M $Zn(OTf)_2$	[22]
**MnO_2/rGO**	5.0	250	1.3	1.23	436	0.1	2M $ZnSO_4$ + 0.1M $MnSO_4$	[23]
**$Mg_{0.26}V_2O_5$•$0.73H_2O$**	5	430	2.12	0.8	Not given	0.05	3M $Zn(CF_3SO_3)_2$	[24]
**$Mn_{0.25}(VO)_{0.75}PO_4$·$2.25H_2O$**	15	207.7	2.81	0.95	Not given	0.1	3M $Zn(CF_3SO_3)_2$	[25]
**MnO_2/3DP CMs**	28.4	282.8	8.04	1.1	226.2	0.1	1M $ZnSO_4$ + 0.1M $MnSO_4$	[26]
**PVO**	2	489	0.98	0.6	Not given	0.1	3M $Zn(CF_3SO_3)_2$	[27]

*: Areal capacity =mass loading × specific capacity

Table S2. EIS impedance values for Zn-S/C and Zn-Te_1S_7/C batteries.

Cell configuration	After cycles			Status
	R_e (Ω)	R_{ct} (Ω)	R_{SEI} (Ω)	
**Zn-S/C**	4.16	25.07	-	Before cycling
	5.04	20.33	9.98	After 1 cycle
	5.24	24.82	15.69	After 2 cycles
	5.81	51.30	404.0	After 5 cycles
**Zn-Te_1S_7/C**	2.32	36.95	-	Before cycling
	3.48	6.71	11.73	After 1 cycle
	4.17	8.78	4.10	After 2 cycles
	4.64	11.43	10.47	After 5 cycles

References

[1] W. Li, K. Wang, K. Jiang, A Low Cost Aqueous Zn–S Battery Realizing Ultrahigh Energy Density, Adv. Sci., 7 (2020) 2000761.

[2] Y. Zhao, D. Wang, X. Li, Q. Yang, Y. Guo, F. Mo, et al., Initiating a Reversible Aqueous Zn/Sulfur Battery through a "Liquid Film", Adv. Mater., 32 (2020) e2003070.

[3] M. Cui, J. Fei, F. Mo, H. Lei, Y. Huang, Ultra-High-Capacity and Dendrite-Free Zinc-Sulfur Conversion Batteries Based on a Low-Cost Deep Eutectic Solvent, ACS Appl. Mater. Interfaces, 13 (2021) 54981-54989.

[4] L.W. Luo, C. Zhang, X. Wu, C. Han, Y. Xu, X. Ji, et al., A Zn-S aqueous primary battery with high energy and flat discharge plateau, Chem Aommun (Camb), 57 (2021) 9918-9921.

[5] A. Amiri, R. Sellers, M. Naraghi, A.A. Polycarpou, Multifunctional Quasi-Solid-State Zinc-Sulfur Battery, ACS Nano, (2022).

[6] D. Liu, B. He, Y. Zhong, J. Chen, L. Yuan, Z. Li, et al., A durable ZnS cathode for aqueous Zn-S batteries, Nano Energy, 101 (2022) 107474.

[7] Z. Xu, Y. Zhang, W. Gou, M. Liu, Y. Sun, X. Han, et al., The key role of concentrated Zn(OTF)($_2$) electrolyte in the performance of aqueous Zn-S batteries, Chem Commun (Camb), 58 (2022) 8145-8148.

[8] M. Yang, Z. Yan, J. Xiao, W. Xin, L. Zhang, H. Peng, et al., Boosting Cathode Activity and Anode Stability of Zn-S Batteries in Aqueous Media Through Cosolvent-Catalyst Synergy, Angew. Chem. Int. Ed. Engl., 61 (2022) e202212666.
[9] H. Zhang, Z. Shang, G. Luo, S. Jiao, R. Cao, Q. Chen, et al., Redox Catalysis Promoted Activation of Sulfur Redox Chemistry for Energy-Dense Flexible Solid-State Zn-S Battery, ACS Nano, 16 (2022) 7344-7351.
[10] T. Zhou, H. Wan, M. Liu, Q. Wu, Z. Fan, Y. Zhu, Regulating uniform nucleation of ZnS enables low-polarized and high stable aqueous Zn–S batteries, Mater. Today Energy, 27 (2022) 101025.
[11] A. Amiri, K. Bashandeh, R. Sellers, L. Vaught, M. Naraghi, A.A. Polycarpou, Fully integrated design of a stretchable kirigami-inspired micro-sized zinc–sulfur battery, J. Mater. Chem. A, (2023).
[12] G. Chang, J. Liu, Y. Hao, C. Huang, Y. Yang, Y. Qian, et al., Bifunctional electrolyte additive with redox mediation and capacity contribution for sulfur cathode in aqueous Zn-S batteries, Chem. Eng. J., 457 (2023) 141083.
[13] Y. Guo, R. Chua, Y. Chen, Y. Cai, E.J.J. Tang, J.J.N. Lim, et al., Hybrid Electrolyte Design for High-Performance Zinc-Sulfur Battery, Small, (2023) e2207133.
[14] J. Li, Z. Cheng, Z. Li, Y. Huang, Rational design of zinc powder anode with high utilization and long cycle life for advanced aqueous Zn-S batteries, Mater Horiz, (2023).
[15] W. Zhang, M. Wang, J. Ma, H. Zhang, L. Fu, B. Song, et al., Bidirectional Atomic Iron Catalysis of Sulfur Redox Conversion in High-Energy Flexible Zn-S Battery, Adv. Funct. Mater., 33 (2023) 2210899.
[16] H. Xu, W. Yang, H. Liu, M. Li, S. Gong, F. Zhao, et al., Boosting kinetics of tellurium redox reaction for high-performance aqueous zinc-tellurium batteries, Chem. Eng. J., 465 (2023) 142896.
[17] Z. Chen, Q. Yang, F. Mo, N. Li, G. Liang, X. Li, et al., Aqueous Zinc-Tellurium Batteries with Ultraflat Discharge Plateau and High Volumetric Capacity, Adv. Mater., 32 (2020) e2001469.
[18] J. Wang, J. Du, J. Zhao, Y. Wang, Y. Tang, G. Cui, Unraveling H($^{+}$)/Zn($^{2+}$) Sequential Conversion Reactions in Tellurium Cathodes for Rechargeable Aqueous Zinc Batteries, J. Phys. Chem. Lett., 12 (2021) 10163-10168.

[19] H. Liu, P. Wu, R. Wang, H. Meng, Y. Zhang, W. Bao, et al., A Photo-rechargeable Aqueous Zinc-Tellurium Battery Enabled by the Janus-Jointed Perovskite/Te Photocathode, ACS Nano, (2023).
[20] H. Wang, W. Yang, H. Xu, M. Li, H. Liu, S. Gong, et al., MoO($_2$) Nanoclusters Embedded in Hierarchical Nitrogen Doped Carbon Nanoflower as Electrocatalytic Mediators in Aqueous Zinc-Tellurium Batteries: Enhancing Electrochemical Kinetics of Tellurium Redox Reaction, Small, (2023) e2304504.
[21] Z. Chen, S. Wang, Z. Wei, Y. Wang, Z. Wu, Y. Hou, et al., Tellurium with Reversible Six-Electron Transfer Chemistry for High-Performance Zinc Batteries, J. Am. Chem. Soc., 145 (2023) 20521-20529.
[22] Y. Du, B. Zhang, W. Zhou, R. Kang, W. Zhang, H. Jin, et al., Laser-radiated tellurium vacancies enable high-performance telluride molybdenum anode for aqueous zinc-ion batteries, Energy Storage Mater., 51 (2022) 29-37.
[23] J. Wang, J.G. Wang, H. Liu, Z. You, Z. Li, F. Kang, et al., A Highly Flexible and Lightweight MnO_2/Graphene Membrane for Superior Zinc-Ion Batteries, Adv. Funct. Mater., 31 (2020) 2007397.
[24] N. Wang, C. Sun, X. Liao, Y. Yuan, H. Cheng, Q. Sun, et al., Reversible (De)Intercalation of Hydrated Zn_{2+} in Mg_{2+}-Stabilized V_2O_5 Nanobelts with High Areal Capacity, Adv. Energy Mater., 10 (2020) 2002293.
[25] J. Guo, W. Ma, Z. Sang, X. Zhang, J. Liang, F. Hou, et al., Low-cost, low-strain and lattice-water-rich $Mn_{0.25}(VO)_{0.75}PO_4\cdot_{2.25}H_2O$ as high-rate and stable cathodes for aqueous Zn-ion batteries, Chem. Eng. J., 428 (2022) 132644.
[26] H. Yang, Y. Wan, K. Sun, M. Zhang, C. Wang, Z. He, et al., Reconciling Mass Loading and Gravimetric Performance of MnO_2 Cathodes by 3D-Printed Carbon Structures for Zinc-Ion Batteries, Adv. Funct. Mater., (2023).
[27] Y. Zhang, Z. Li, M. Liu, J. Liu, Construction of novel polyaniline-intercalated hierarchical porous V_2O_5 nanobelts with enhanced diffusion kinetics and ultra-stable cyclability for aqueous zinc-ion batteries, Chem. Eng. J., 463 (2023) 142425.
